# Thromboprophylaxis in elective spinal surgery: A protocol for systematic review

**DOI:** 10.1097/MD.0000000000020127

**Published:** 2020-05-22

**Authors:** María J. Colomina, Joan Bagó, Javier Pérez-Bracchiglione, Maria Betina Nishishinya Aquino, Karla R. Salas, Carolina Requeijo, Gerard Urrútia

**Affiliations:** a Department of Anaesthesiology and Intensive Care, Hospital Universitari Bellvitge, L’Hospitalet de LLobregat, Barcelona, Universitat de Barcelona; b Spine Surgery Unit, Department of Orthopedic and Trauma Surgery, Hospital Universitari Vall d’Hebron, Barcelona, Universitat Autònoma de Barcelona, Barcelona, Spain; c Interdisciplinary Centre for Health Studies (CIESAL), Universidad de Valparaíso, Chile; d Iberoamerican Cochrane Centre, Sant Pau Biomedical Research Institute (IIB Sant Pau); e Iberoamerican Cochrane Centre, Sant Pau Biomedical Research Institute (IIB Sant Pau), CIBERESP, Barcelona, Spain.

**Keywords:** chemoprophylaxis, deep vein thrombosis, epidural hematoma, mechanoprophylaxis, pulmonary embolism, spine surgery, thromboprophylaxis

## Abstract

**Background:**

Venous thromboembolism (VTE) is a serious, sometimes life-threatening complication that can occur following spine surgery. The incidence of VTE, and the optimal type and timing of thromboprophylaxis for this complication in elective spine surgery is a matter of debate.

**Objective:**

To perform a systematic review with the aim of clarifying the efficacy and adverse effects of mechanical and chemical prophylaxis for preventing thromboembolic complications in elective spine surgery for conditions other than trauma and malignant disease.

**Methods/design:**

A search strategy of related articles up to March 2018 was designed and executed in Medline and Embase. Patients: adolescents (>10 years) and adults undergoing elective surgery for spinal deformity or degenerative disease (from C1 to S1). Intervention: Perioperative mechanical and chemical thromboprophylaxis. Studies could be randomized controlled trials or observational studies that reported data on any relevant clinical outcomes.

**Results:**

In total, 2451 uniquecitations were identified and 35 studies were ultimately included in the systematic review. The overall mean incidence of complications was 3.7% for deep venous thrombosis, 0.0% for pulmonary embolism, and 3.7% for bleeding in chemoprophylaxis group; 2.9% for deep venous thrombosis, 0.4% for pulmonary embolism and 0.0% for bleeding in mechanoprophylaxis; and 0.7% for deep venous thrombosis, 0.1% for pulmonary embolism and 0.2% for bleeding in mixed prophylaxis group with no specific data on these rates for the type of patient and type and location of surgery. None of the articles retrieved provided information on the adolescent population.

**Discussion and Conclusions:**

The poor design and high variability among the studies regarding characteristics of study population, details of interventions, and definitions of outcomes, determines a low quality of the available evidence and limits the interpretation of the results. We were unable to identify a clear advantage of one type of thromboprophylaxis over the other, although there was an increased risk of bleeding with chemoprophylaxis, which could favor the use of mechanoprophylaxis in this scenario.

## Abstract 2

Supplemental Digital Content is available in the text

**Highlights**

- There is no clinically difference between chemoprophylaxis and mechanoprophylaxis regarding VTE or PE incidence.
- The appropriate time for the start of postoperative anticoagulation has not yet been determined.
- The use of chemoprophylaxis is related to the occurrence of bleeding complications.
- There is a lack of information in the adolescent population (under 18 years of age) on the incidence of VTE or PE or the effectiveness of thromboprophylaxis.
- This systematic review confirms the low quality of research in this field.

## 1 Introduction

Venous thromboembolism (VTE), including deep venous thrombosis (DVT), and pulmonary embolism (PE), is a serious and potentially fatal complication that can occur in the postoperative period following spine surgery.^[1]^ DVT and PE have both been extensively studied in several types of orthopedic and trauma surgeries. However, data on the incidence and impact of VTE in spine surgery is more limited, and currently, there is no clear consensus on what constitutes optimal thromboprophylaxis in these procedures.^[2]^ This is particularly true for elective spine surgery performed for conditions other than trauma or a malignant disease.^[1,3,4]^

The documented incidence of VTE in spine surgery varies widely from 0.3% to 31%, depending on the population studied and the methods used to detect this complication.^[5]^ It is likely that this considerable divergence is the result of including patients with different conditions (e.g., degenerative spinal disease, deformity, traumatic injury, and tumors) affecting different parts of the spine and treated with heterogeneous surgical techniques.^[6,7]^

The risk–benefit relationship of thromboprophylaxis in this setting should also be considered. When deciding on the type and timing of thromboprophylaxis, the risk of postoperative bleeding and epidural hematoma (EDH) must be carefully weighed against the benefits of averting thromboembolic complications, and this may be a complex task in clinical practice.^[7–11]^

Among the systematic reviews on the indications and risks of thromboprophylaxis in general spine surgery^[5,12–14]^ we highlight the study by Glotzbecker et al,^[5]^ which reported a DVT rate influenced by the method of thromboprophylaxis—no prophylaxis, 2.7%; compression stockings (CS), 2.7%; sequential pneumatic compression devices (SPC), 4.6%; SPC + CS, 1.3%; chemical prophylaxis 0.6%; and vena cava filter with/without another prophylaxis method, 12%—and by the diagnostic method used, with a range of 1% to 12.3%. The authors of this review concluded that the DVT rate was quite low in elective spine surgery and that mechanical prophylaxis could be the method of choice in most cases. Routine chemoprophylaxis was not recommended because of insufficient evidence, except in patients with cord lesions and those undergoing high-risk spinal surgery (simultaneous dual approach or malignant disease). The systematic review by Cheng et al^[12]^ was mainly focused on the risk of anticoagulant administration in general spine surgery (mechanical thromboprophylaxis was not assessed). The studies included were grouped according to the type of spine disease: degenerative, deformity, trauma, infection, and malignant disease. There was a higher risk of DVT in spine surgery for non-traumatic causes (6%, range 0–19%) and for deformity (5.3%, range 2–14%), and a lower risk in surgery for degenerative conditions (2.3%, range 0–9%). The overall risk of DVT in elective surgery without pharmacological prophylaxis was 1% to 2%. The risk of major bleeding ranged from 0% to 4.3% with use of a variety of anticoagulant agents. This study was unable to establish the safest time point to initiate coagulation in the postoperative period, as none of the studies had defined this objective, nor the most appropriate treatment option. Some of the conclusions were derived from a small number of studies; hence, the authors considered that the quality of the evidence for the associated risk of DVT in the different types of spine surgery was of low or very low level, particularly in the case of elective surgery. Despite these limitations, the conclusions suggested that patients undergoing elective spine surgery could undergo the procedure without the use of pharmacologic prophylaxis. A more recent systematic review carried out by Oliveira et al^[13]^ reported average overall rates of 3.55% and 1.04% for DVT and PE, respectively, in all types of spine surgery. The authors stated that it was not possible to indicate a standard thromboprophylaxis for spine surgery, as can be done for hip and knee surgeries, because of the heterogeneous nature of the studies. Additionally, chemoprophylaxis may not be safe due to the possibility of bleeding complications. Therefore, it falls to the spinal surgeon to analyze the risk of DVT/PE individually for each specific case. The authors emphasized the importance of symptomatic EDH, which often shows rapidly progressing neurological deterioration and can be associated with coagulopathy induced by the administration of anticoagulants. Around 4% of patients who received systemic LMWH prophylaxis developed bleeding complications, compared with the 0.5% to 2.5% of patients who developed DVT following spinal surgery, making this a substantial disincentive for chemoprophylaxis in these procedures, although this was not specifically described in the review.^[15,16]^

On the other hand, the North American Spine Society (NASS) and American College of Chest Physicians (ACCP) have published guideline for VTE prophylaxis in non-traumatic, non-neoplastic elective spine surgery.^[17,18]^ Nevertheless, all these recommendations are based on a small number of studies including a limited number of patients and showing significant methodological defects.

Since the publication of these guidelines, new data on the outcomes of different types of thromboprophylaxis in spinal surgery have been published. As there is considerable uncertainty regarding the optimal thromboprophylactic regimen to use in elective spine surgery, we carried out a systematic review on this topic. We aimed to evaluate the efficacy and safety of mechanical prophylaxis, chemical prophylaxis or both for preventing VTE in adolescents and adults undergoing elective spine surgery for conditions other than trauma or malignant disease.

## 2 Methods

We conducted a systematic review following the methodology outlined in the Cochrane Handbook for Systematic Reviews of Interventions.^[19]^ We searched EMBASE and PubMed, from inception to March 2018. Details of search strategy are provided in Appendix 1. We also manually checked the reference lists of all relevant studies that were identified by the above mentioned searches, as well. Only studies published as a full report were included. In order to identify other potentially relevant primary studies, we also reviewed the references of other systematic reviews and clinical practice guidelines referring to thromboprophylaxis in spinal surgery.

As inclusion criteria, we considered randomized controlled trials (RCTs) and observational studies (minimum of 30 participants), whether controlled or not, that included adolescent (>10 years old) and/or adult patients undergoing elective surgery for deformities and/or degenerative spinal disease, from C1 to S1. We excluded studies including other spinal conditions, if these represented >50% of the total study population and specific data for the relevant population was not available.

Studies had to evaluate, either alone or in combination, a mechanical (CS or SPC), or chemical (low molecular weight heparin [LMWH], low-dose unfractionated heparin [LDUH], aspirin, or a new direct oral anticoagulants) thromboprophylaxis. Prophylaxis could be administered either pre, intra, and/or postoperatively. We did not consider vena cava filter for purposes of this systematic review. Outcomes of interest were DVT, PE, and risk of bleeding or EDH. We excluded studies in language other than English or Spanish.

Two authors (GU and BN) independently screened all articles based on title and abstract. Disagreements were resolved through discussion. Potentially relevant studies were obtained in full text, and again, were independently assessed for inclusion by the same two authors. A third author was contacted (MJC) when disagreement persisted.

One author (KS or CR) extracted data from each study using a predefined data extraction form, which included information related to characteristics of the population, interventions and definition of the outcome measures, details of design and results. A second author (JPB or GU) cross-checked all the extracted data. Two authors (KS or CR or JBP or GU) assessed the risk of bias of included RCTs using the Cochrane Risk of Bias tool.^[19]^

For data analysis, we grouped studies into three categories: chemoprophylaxis, mechanical, and mixed thromboprophylaxis. We intended to compare the effectiveness between each category using random-effects meta-analysis were possible. Otherwise, results would be presented descriptively. As we anticipated to find many single arm observational studies, we also planned to combine incidence rates across studies for each category.

## 3 Results

**Figure 1:**
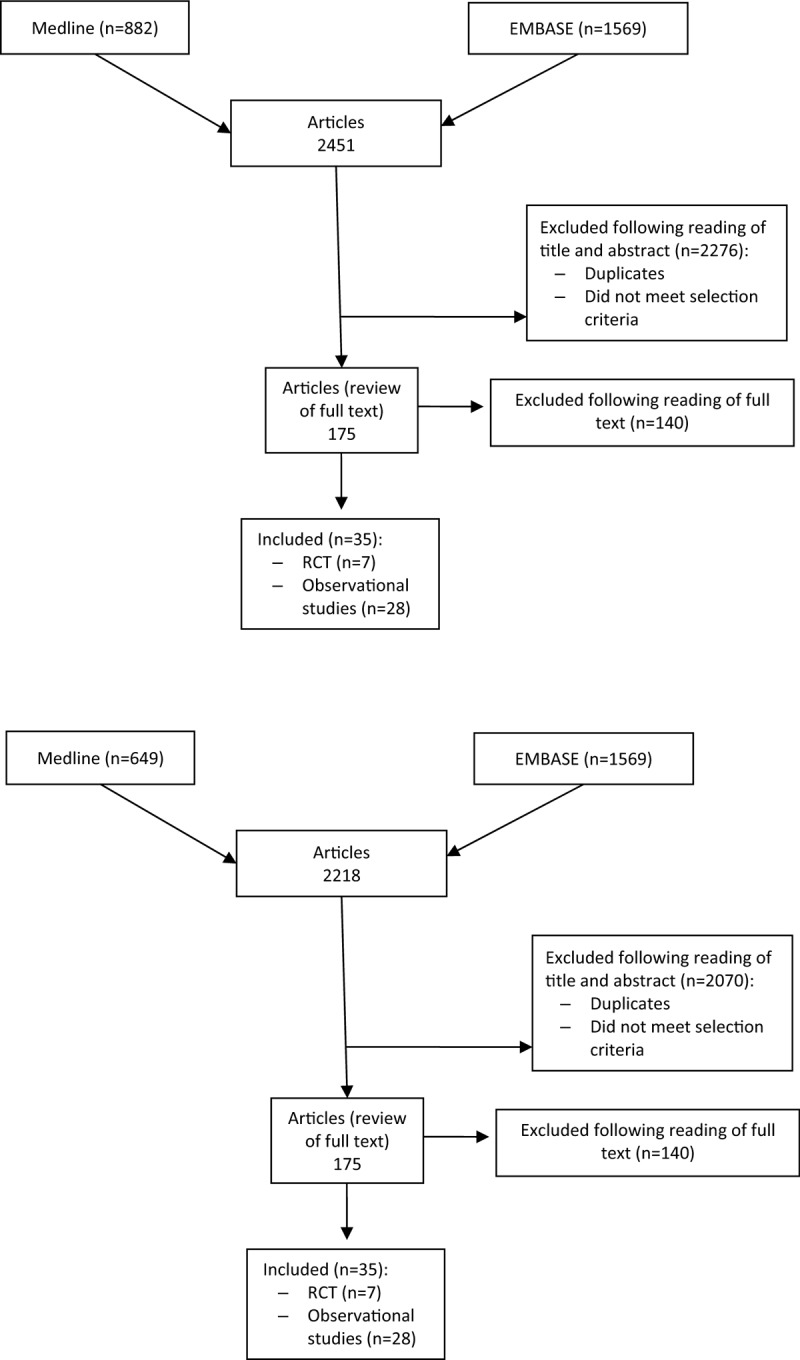
Flow chart showing eligibility of studies.

The initial search strategy retrieved 2451 unique references. After the screening process, 35 studies met eligibility criteria, including 7 RCTs^[11,20–25]^ and 28 observational studies^[3,6,8,26–50]^ (Fig. 1). In Appendix 2 we provide details of the included studies. We also reviewed the references list from 8 systematic reviews,^[5,12–14,51–54]^ and two clinical practice guidelines,^[17,55]^ with no additional relevant primary studies identified. Appendix 3 provides a list of excluded studies. Overall, most of the included RCTs showed an unclear risk of selection bias, and a high risk of detection and performance bias (Appendix 4). Regarding the observational studies, we considered that their overall quality was low since either they used historical controls or they were a single arm study, mostly retrospective. In addition, there was great clinical variability in terms of study population, type of surgery, methods of thromboprophylaxis, definition of events, and/or VTE diagnostic methods among them.

Considering the high clinical heterogeneity among all the included controlled studies, we decided not to perform a meta-analysis of comparative effectiveness. Instead, we performed a pooled analysis of the incidence rate of the events within each group. We provide a narrative synthesis of the comparative effects of different methods of thromboprophylaxis in Table 1.

**Table 1:**
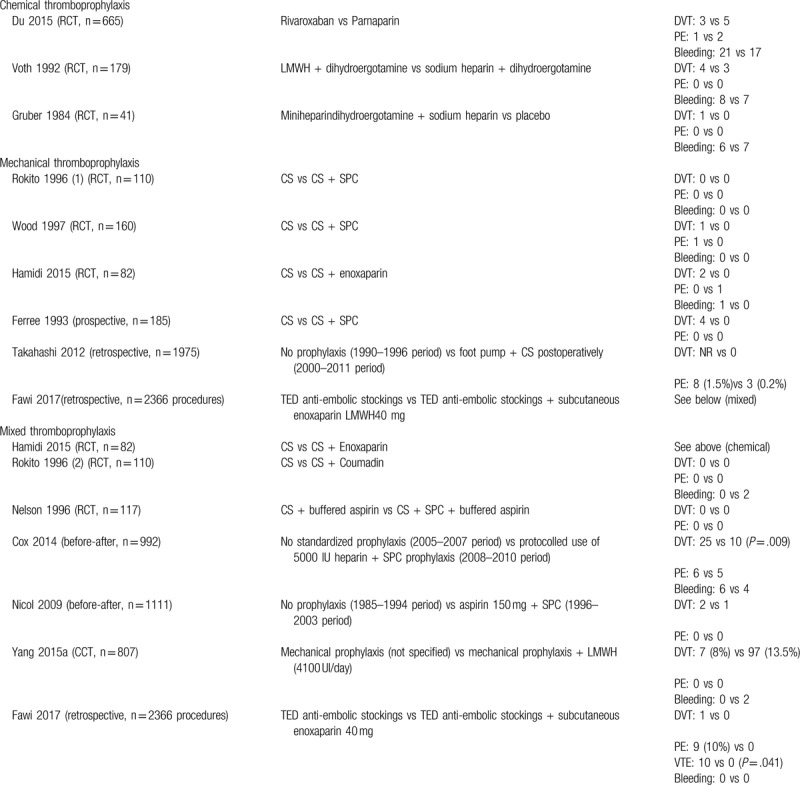
Summary of the studies of comparative effectiveness.

## 4 Effects on VTE and bleeding according to the type of prophylaxis

### 4.1 Chemical prophylaxis (n = 6)

Three RCTs and three single-arm observational studies were included in this group. Two RCTs compared active pharmacologic prophylactic agents with each other (rivaroxaban vs parnaparin, and LMWH + dihydroergotamine vs sodium heparin + dihydroergotamine),^[20,23]^ while the other compared miniheparin dihydroergotamine + sodium heparin vs placebo,^[25]^ showing no significant differences between the groups for thromboembolic events (DVT and PE) or for bleeding/EDH. The three observational studies evaluated chemical prophylaxis with dalteparin^[3]^ or LMWH.^[41,49]^

The overall incidence (pooled effect across all chemoprophylaxis arms [n = 8]) of DVT in these studies was 3.7% (0.0–13.1), 0.0% (0.0–0.0) for PE, and for bleeding complications 3.7% (0.7–8.6) (Fig. 2  A–C). In the study by Du et al^[20]^ (rivaroxaban or parnaparin groups) only 3 events out of 38 reported consisted of serious bleeding events. In this study, there was also 1 case (0.3%) of EDH in the rivaroxaban group and 1 death due to incoercible gastrointestinal bleeding in the parnaparin group. Only one more case of EDH was observed in the study by Yang et al,^[49]^ which implies an overall EDH rate of 0.10% (2 events in 2.062 patients) in this group.

**Figure 2:**
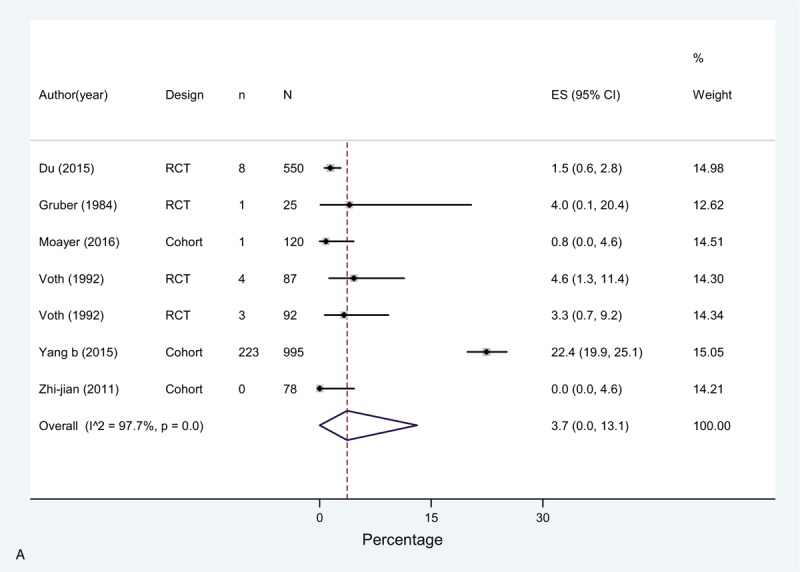
(A) Meta-analysis of incidence of DVT in Chemoprophylaxis studies. (B) Meta-analysis of incidence of PE in Chemoprophylaxis studies. (C) Meta-analysis of incidence of bleeding in Chemoprophylaxis studies.

**Figure 2 (Continued):**
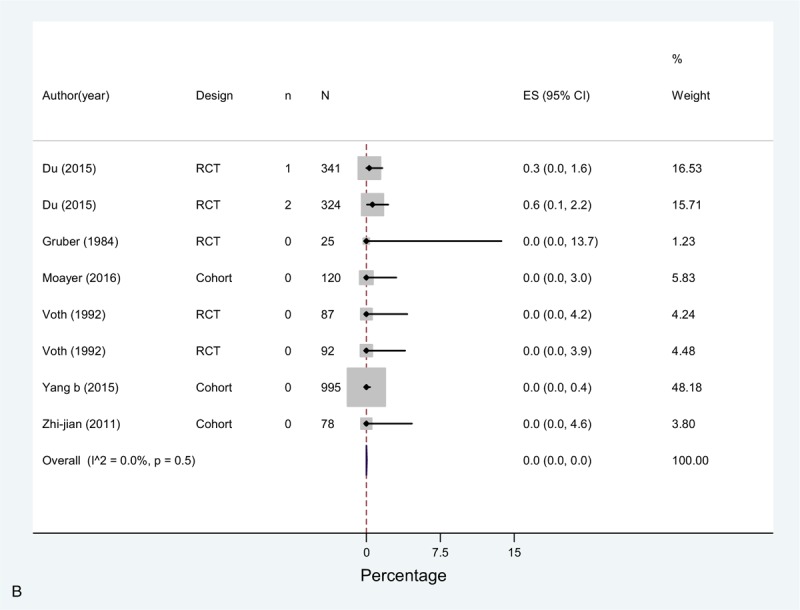
(A) Meta-analysis of incidence of DVT in Chemoprophylaxis studies. (B) Meta-analysis of incidence of PE in Chemoprophylaxis studies. (C) Meta-analysis of incidence of bleeding in Chemoprophylaxis studies.

**Figure 2 (Continued):**
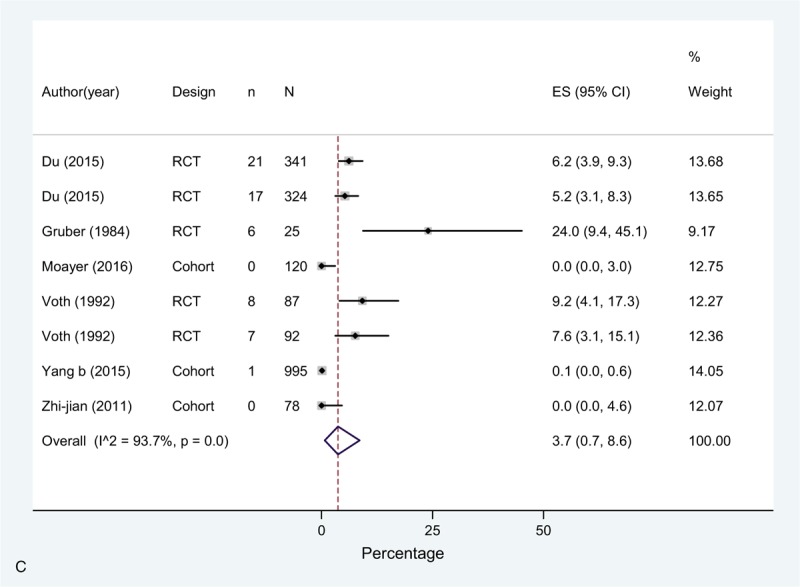
(A) Meta-analysis of incidence of DVT in Chemoprophylaxis studies. (B) Meta-analysis of incidence of PE in Chemoprophylaxis studies. (C) Meta-analysis of incidence of bleeding in Chemoprophylaxis studies.

The meta-analysis showed high heterogeneity across studies for DVT, due to a high incidence reported by Yang et al^[49]^ of 22.4% in contrast with all the rest of the studies, and bleeding, where in the study by Gruber et al^[25]^ 24% of the patients in the chemoprophylaxis arm presented a bleeding event defined as increased intraoperative bleeding, similar to Voth et al^[23]^ (overall rate was 8.4% for both study groups).

### 4.2 Mechanical prophylaxis (n = 19)

Three RCTs and 16 observational studies (four of them controlled) provided data for this group, including arms with CS, CS + SPC, and foot pump during surgery + CS. Three out of the seven controlled studies compared CS vs CS + SPC, with few more VTE events in the CS alone group.^[8,11,24]^ Two more studies compared CS vs CS + enoxaparin which found comparable^[21]^ or better^[48]^ efficacy results with no increase in bleeding risk with the use of enoxaparin. One more study compared no thromboprophylaxis vs foot pump + CS with better results with the later.^[38]^ One more study compared mechanical prophylaxis (not specified) vs mixed,^[43]^ with better results in favor of the mixed thromboprophylaxis.

Twelve single arm observational studies assessed a variety of mechanical prophylaxis, including CS + SPC,^[6,26,29,32,36,39]^ SPC,^[30,31]^ CS or SPC,^[45]^ foot pump,^[44]^ and mechanical (not specified).^[43]^

The overall incidence (pooled effect across all mechanical prophylaxis arms [n = 23]) of DVT was 2.9% (1.2–5.2%) and 0.4% (0.1–0.8%) for PE. Bleeding complications had a 0.0% incidence (Fig. 3  A–C). Of note, only 10 studies accounting for 12 study arms reported this last outcome with very few events.

**Figure 3:**
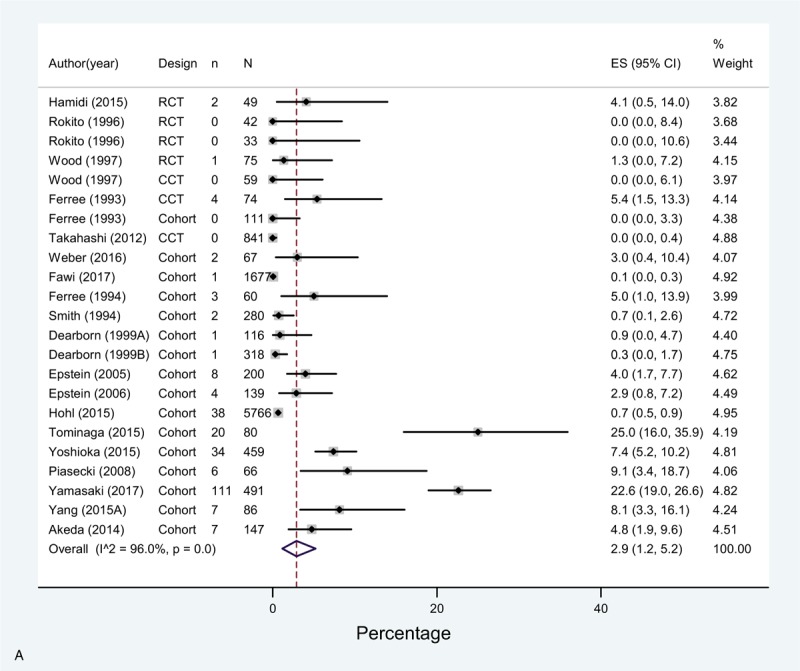
(A) Meta-analysis of incidence of DVT in Mechanoprophylaxis studies. (B) Meta-analysis of incidence of PE in Mechanoprophylaxis studies. (C) Meta-analysis of incidence of bleeding in Mechanoprophylaxis studies.

**Figure 3 (Continued):**
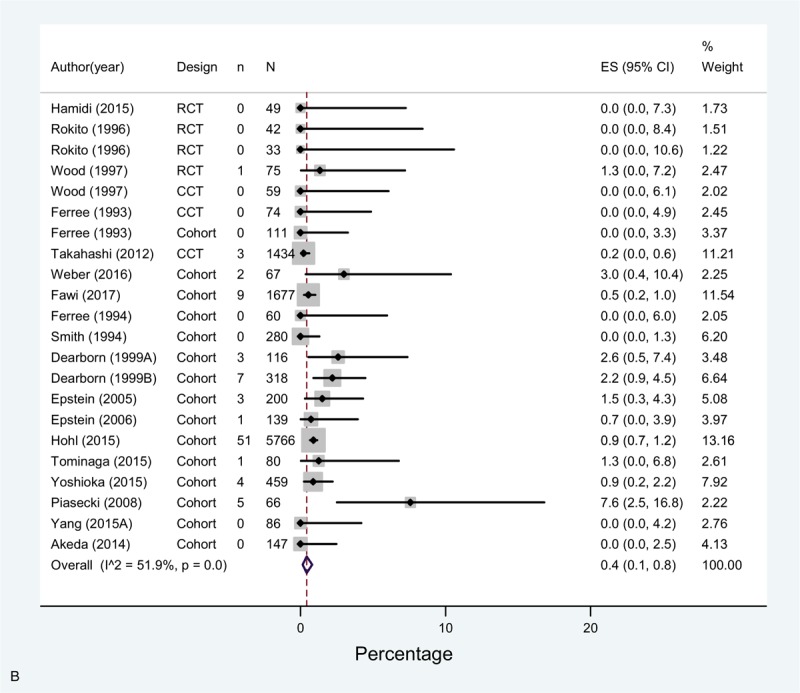
(A) Meta-analysis of incidence of DVT in Mechanoprophylaxis studies. (B) Meta-analysis of incidence of PE in Mechanoprophylaxis studies. (C) Meta-analysis of incidence of bleeding in Mechanoprophylaxis studies.

**Figure 3 (Continued):**
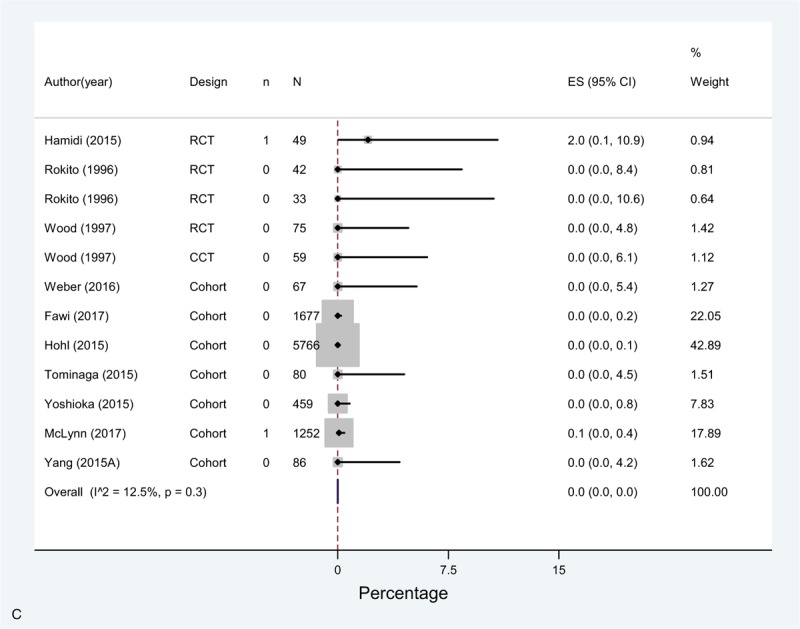
(A) Meta-analysis of incidence of DVT in Mechanoprophylaxis studies. (B) Meta-analysis of incidence of PE in Mechanoprophylaxis studies. (C) Meta-analysis of incidence of bleeding in Mechanoprophylaxis studies.

Similar to chemoprophylaxis, the meta-analysis showed high heterogeneity across studies for DVT and moderate for PE. DVT rates varied across studies between 0% in 5 study arms^[8,11,24,38]^ and 23.8% in Tominaga 2015,^[39]^ or 22.6% in Yamasaki 2017.^[39,45]^ As for PE, reported rates varied between 0% and 3% in all studies, except for Piasecki 2008, that reported 7.6% incidence.^[44]^ As for bleeding, the estimates across studies were consistent, and the overall risk was lower than that with chemoprophylaxis.

### 4.3 Mixed prophylaxis: chemical–mechanical (n = 15)

Three RCT and 12 observational studies (five of them controlled) provided data for this group. Two of the RCT compared mixed with mechanical thromboprophylaxis^[11,21]^ with similar results, although in Rokito^[11]^ mechanical prophylaxis proved to be safer than mixed prophylaxis in terms of bleeding. Another RCT compared two modes of same mixed thromboprophylaxis,^[22]^ with similar results. As for the controlled observational studies, two studies compared mixed against no prophylaxis, with better results for mixed thromboprophylaxis.^[28,33]^ The other three observational studies compared mixed vs mechanical thromboprophylaxis,^[43,46,48]^ with better results in favor of mixed thromboprophylaxis. Overall, there was a slight increase in bleeding risk in the mixed group.

The other seven single arm studies^[27,34,35,37,42,47,50]^ evaluated a variety of mixed interventions: nadroparin + CS,^[42]^ LMWH + CS,^[27,35]^ warfarin or LMWH + CS + SPC,^[34]^ LMWH + SPC,^[37]^ argatroban or LMWH + SPC,^[50]^ and UH or LMWH or warfarin + SPC.^[47]^

The overall incidence (pooled effect across all mixed prophylaxis arms [n = 14]) of DVT in these studies was 0.7% (0.0–2.5), 0.1% (0.0–0.6) for PE, and for bleeding complications 0.2% (0.1–0.5) (Fig. 4  A–C).

**Figure 4:**
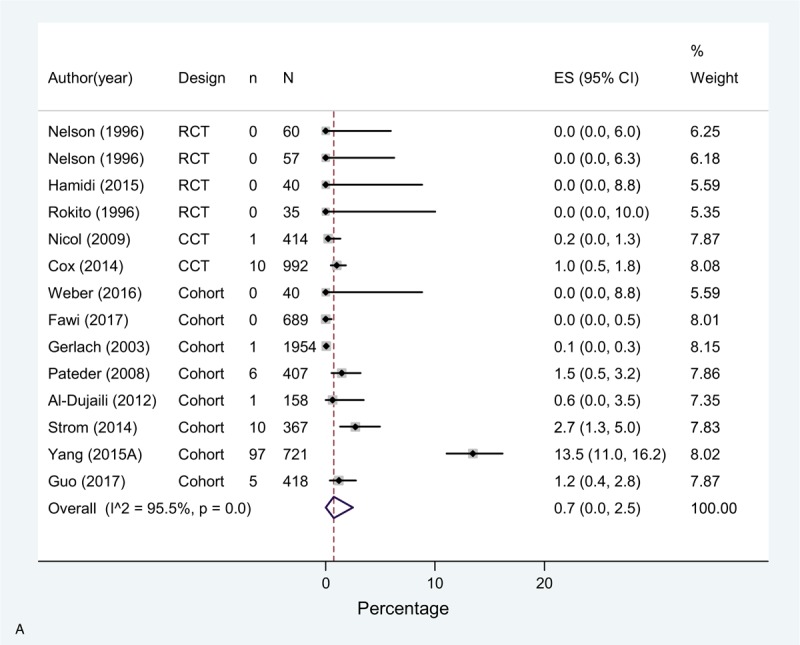
(A) Meta-analysis of incidence of DVT in mixed prophylaxis studies. (B) Meta-analysis of incidence of PE in mixed prophylaxis studies. (C) Meta-analysis of incidence of bleeding in mixed prophylaxis studies.

**Figure 4 (Continued):**
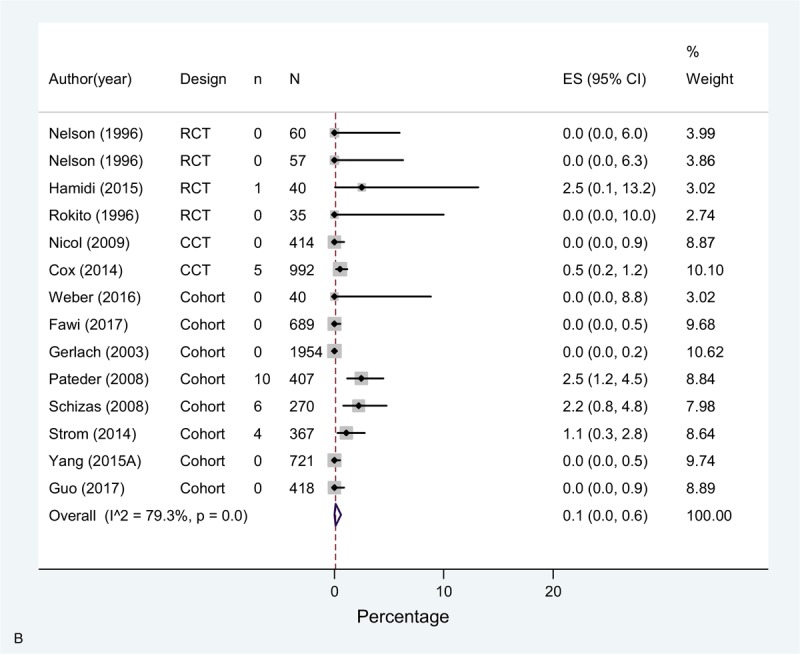
(A) Meta-analysis of incidence of DVT in mixed prophylaxis studies. (B) Meta-analysis of incidence of PE in mixed prophylaxis studies. (C) Meta-analysis of incidence of bleeding in mixed prophylaxis studies.

**Figure 4 (Continued):**
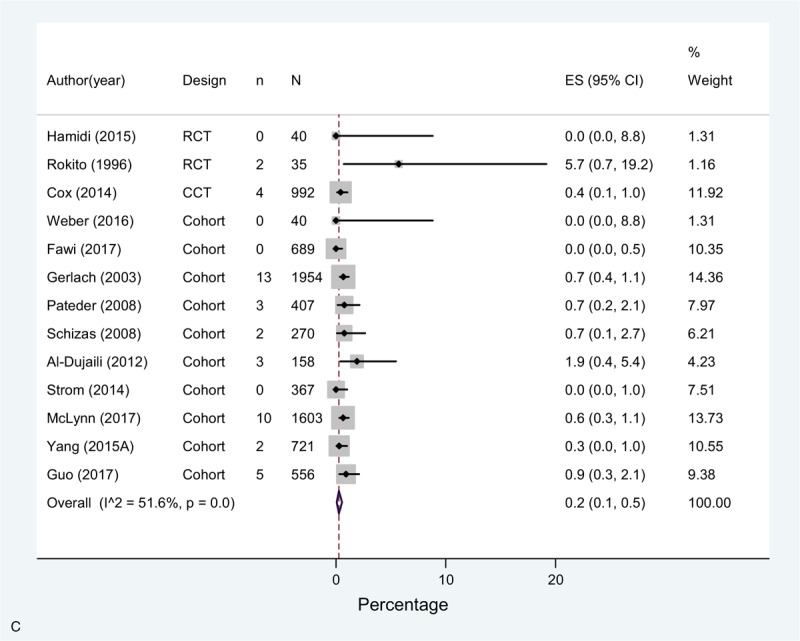
(A) Meta-analysis of incidence of DVT in mixed prophylaxis studies. (B) Meta-analysis of incidence of PE in mixed prophylaxis studies. (C) Meta-analysis of incidence of bleeding in mixed prophylaxis studies.

A high statistical heterogeneity was observed for DVT and PE estimates across studies, and moderate for bleeding. Regarding DVT, most of studies showed an incidence of <3%, except for one study using LWMH + mechanical prophylaxis (not specified), that showed an incidence of 13.5%.^[43]^ As for PE, two single arm studies (one comparing warfarin or LMWH + CS + SPC^[34]^ and the other comparing LMWH + SC ^[35]^) and one study arm of a RCT with enoxaparin + CS^[21]^ showed higher rates compared to the rest of studies.

The studies showed considerable clinical heterogeneity in the populations included, types of surgery, and other important factors related to potential bias.

Regarding EDH risk, the study by Gerlach et al^[42]^ reported 13 (0.7%) cases of postoperative EDH. In 5 of them, the hematoma occurred before nadoparin administration. Four patients with EDH died (0.2%) and 4 others had a persistent neurological deficit.

In the study by Cox et al,^[28]^ 4 patients presented symptomatic HED requiring surgical evacuation after implantation of a thromboprophylaxis protocol with heparin.

In the study by Al-Dujaili et al,^[27]^ 3 cases of EDH occurred (1.8%), all in patients who had received pharmacologic prophylaxis. One patient required decompression surgery and hematoma evacuation, with posterior recovery. None of them had residual neurological deficits.

Other EDH events were observed in the study by Pateder et al^[34]^ (3 cases detected in patients receiving warfarin), Guo et al^[50]^ (1 case receiving LMWH), Schizas et al^[35]^ (two cases of postoperative hematomas, without development of neurological symptoms or signs, requiring emergency evacuation), and Yang et al^[49]^ (two cases). Overall, the inside of EDH in this group was of 0.30% (33 events in 10.933 patients).

## 5 Discussion

Our results show that the mean incidence of DVT and PE is 3.7% and 0%, respectively in the chemoprophylaxis group; 2.9% and 0.4% in the mechanoprophylaxis group; and 0.7% and 0.1% in the mixed prophylaxis group. In the case of bleeding, the mean incidence was 3.7%, 0%, and 0.2% for chemoprophylaxis, mechanoprophylaxis, and mixed prophylaxis, respectively. We were unable to obtain specific rates according to the type of patient, type of surgery or spinal area, based on the available data.

The aim of this systematic review was to provide evidence for clinicians seeking to establish optimum thromboprophylaxis for the specific population of adolescent and adult patients undergoing elective surgery of the spine. Contrary to other reviews on this topic, we did not include studies or study arms with patients who had not received any type of thromboprophylaxis in our analysis. In our opinion, thromboprophylaxis is a standard of care and the interest lies in determining which is best, not in comparing thromboprophylaxis with no treatment. Glotzbecker et al^[5]^ published a systematic review including elective, trauma and tumour surgery of the spine, where DVT incidence was 4.6% for mechanical prophylaxis and 0.6 for combined treatment, and PE incidence was 1.1% for mechanical and 0.3% for combined prophylaxis. In our opinion, a major limitation of this review is the inclusion of patients with different diseases and a diversity of surgeries. This adds confusion to the interpretation of the findings. Oliveira et al^[13]^ conducted a systematic using as search keywords “spine” and “thrombosis.” They reported and overall rates of 3.55% and 1.04% for DVT and PE, respectively, in all types of spine surgery. This review presents obvious methodological flaws that seriously limit its validity. Recently, Moshental et al^[14]^ conducted a systematic review and meta-analysis, whose aim was to determine the incidence of DVT and PE in spine surgery (not including pediatric, trauma nor neoplasic cases) in patients receiving no thromboprophylaxis, mechanical prophylaxis, and chemical prophylaxis. They found a higher incidence of these events in the mechanical prophylaxis group (DVT: 1%, PE: 0.81%) compared to the chemical prophylaxis group (DVT: 0.85%, PE: 0.58%) but the difference was not statistically significant. The incidence of DVT/PE reported in this review is somewhat lower than that reported in our study. As the authors recognize, this may be explained by the fact that they only included symptomatic DVT studies whereas in our review we included DVT diagnosed with different techniques. In addition, our review included more studies and, unlike Moshental et al, we excluded groups of patients who did not receive any thromboprophylaxis. From the point of view of safety, it does not currently seem appropriate to provide any type of thromboembolic prophylaxis in this type of surgery. In contrast to the study by Moshental et al,^[14]^ which contained a meta-analysis only of the incidence of complications, our review also narratively describes the results of comparative studies, and provides a more detailed description of each included study.

Thromboprophylaxis in spine surgery is a standard of care but evidence is lacking to prove which is the best method of treatment. Since the first systematic review by Glotzbecker et al in 2009,^[5]^ research on this topic has not been able to provide relevant information to resolve this question. This opinion coincides with the view set forth in the North American Spine Society guidelines, which pointed out that evidence on the risks or benefits of chemical prophylaxis for VTE in elective spine surgery is insufficient to formulate recommendations.^[17]^ In ACCP 9th edition (2012) guidelines,^[18]^ within the recommendations for patients undergoing spine surgery with no risk factors, mechanical prophylaxis, preferably with SPC is recommended over no prophylaxis (Grade 2C), unfractionated heparin (Grade 2C), or low-molecular-weight heparin (Grade 2C), without specifying the spinal segment treated or the type of surgery. In patients at a high risk for VTE undergoing spine surgery (including those with malignant disease and those treated with a combined anterior–posterior approach), the authors suggest adding chemoprophylaxis medication to mechanical prophylaxis once adequate hemostasis is established and the risk of bleeding decreases (Grade 2C). In this guide, the need to use some kind of thromboprophylaxis is clearly stated. In addition, the NICE CG92 guidelines^[56]^ followed this line, stating that patients undergoing spine surgery should be evaluated on an individual basis to weight the risk of bleeding against that of VTE because of the considerable heterogeneity of this population as a risk group. This document also mentioned that the appropriate time for the start of postoperative anticoagulation and the type (mechanical or chemical) has not yet been determined.

A peculiar feature of spine surgery is the risk of perioperative bleeding and/or EDH. Many authors have taken a cautious stance with regard to routine use of chemical prophylaxis in spine surgery for conditions other than trauma and malignant disease^[48–52]^ because a larger incidence of postoperative bleeding complications has been observed in relation to chemical prophylaxis (3.7%) than combined prophylaxis (0.2%).^[2]^ In our review, the overall incidence of postoperative complications related to surgical wound bleeding and postoperative blood loss was 3.7% in chemoprophylaxis group, 0.0% in mechanical group and 0.2% in mixed group. The incidence of EDH was globally low; differences between the different types of thromboprophylaxis were not striking (0.1 chemical, 0% mechanical, and 0.3% combined). Our findings are overlapping with those reported by other authors. Mosenthal et al^[14]^ found an EDH rate of 0.3% but no data on bleeding complications were gathered. Cheng et al^[12,14]^ conducted a systematic review focused on the risk of anticoagulant administration in spine surgery (mechanical thromboprophylaxis was not assessed). The risk of major bleeding ranged from 0% to 4.3% with use of a variety of anticoagulant agents. However, they could not establish a clear relationship between the risk of bleeding and the administered agent.

As a strength of our study, the search strategy included not only databases but also a review of references of previous related SR. Also, a detailed description of included primary studies, and a meta-analysis of incidence of events for each type of thromboprophylaxis. Among the limitations, 18 observational non-controlled studies were included. The remaining 10 studies included a control group, either randomized or not, but the heterogeneous comparisons precluded to pool comparative data. Some of the studies were old (publication range 1984–2018), either retrospective and prospective. The inclusion criteria were often poorly defined (population, interventions) and the populations were heterogeneous. The baseline risk for VTE and the timing of the prophylaxis and follow-up (at hospital discharge, 1 week, 1 month) were not clearly specified in some studies and were very diverse in others.

This systematic review confirms the low quality of research in this field. However, some findings seem unquestionable. First, there is a complete lack of information in the adolescent population (under 18 years of age) on the incidence of VTE or PE or the effectiveness of thromboprophylaxis in this age group. Consequently, no recommendation is possible. Second, the use of chemoprophylaxis is related to the occurrence of bleeding complications. And third, there is no evidence of any clinically relevant difference between chemoprophylaxis and mechanoprophylaxis regarding VTE or PE incidence. This information must be kept in mind by surgeons when deciding on the most appropriate thromboprophylaxis for each patient. These data would probably favor the use of mechanoprophylaxis in uncomplicated, non-traumatic non-tumoral surgery, especially when the procedure may require spinal canal opening. Nonetheless, there is a need for additional, well-designed comparative studies to establish high-level evidence for the safety and effectiveness of this practice Appendix 5.

## Acknowledgments

All data generated or analyzed during this study are included in this article.

## Author contributions

Drs MJC, JB, MBNA, and GU contributed to conception, design, data analysis, and writing the manuscript. Drs GU, MBNA, JPB, KRS, and CR contributed in literature search, data collection and editing the manuscript. Drs GB and KK contributed to the editing and revision of the manuscript.

## Supplementary Material

Supplemental Digital Content

## Supplementary Material

Supplemental Digital Content

## Supplementary Material

Supplemental Digital Content

## Supplementary Material

Supplemental Digital Content

## Supplementary Material

Supplemental Digital Content
