# Supplementary material for: Thromboprophylaxis in elective spinal surgery: A protocol for systematic review
Source: Medicine (Baltimore). 2020 May 22;99(21):e20127. doi: 10.1097/MD.0000000000020127 (PMC7249943; doi:10.1097/MD.0000000000020127)
Supplement: Supplemental Digital Content [file medi-99-e20127-s001.docx]

**Appendix 1. Search strategy**

| **MEDLINE**  PubMed  26/03/2018 | #1 “Spinal Diseases”[Mesh] 111478  #2 “Spine/surgery”[Mesh] 33977  #3 “Spinal Nerves/surgery”[Mesh] 11364  #4 “Spinal Fusion”[Mesh] 21662  #5 spine[tiab] 106826  #6 spinal[ti] 114517  #7 spinal stenos*[tiab] 4321  #8 spinal fusion[tiab] 5827  #9 spondylitis[tiab] 15891  #10 spondylosis[tiab] 3057  #11 scoliosis[tiab] 18223  #12 kyphosis[tiab] 7324  #13 kyphoscoliosis[tiab] 1495  #14 lordosis[tiab] 5790  #15 lumbar fusion[tiab] 1745  #16 #1 OR #2 OR #3 OR #4 OR #5 OR #6 OR #7 OR #8 OR #9 OR #10 OR #11 OR #12 OR #13 OR #14 OR #15 311152  #17 "Venous Thromboembolism"[Mesh] 8049  #18 "Venous Thrombosis"[Mesh] 51009  #19 "Pulmonary embolism"[Mesh] 35925  #20 thrombus*[tiab] 34295  #21 thrombotic*[tiab] 34125  #22 thrombolic*[tiab] 42  #23 thromboemboli*[tiab] 53560  #24 thrombos*[tiab] 135779  #25 thromboph*[tiab] 14218  #26 deep vein thromb*[tiab] 14820  #27 deep vein embol*[tiab] 13  #28 deep*[ti] AND vein*[ti] AND thromb*[ti] 5362  #29 deep*[ti] AND vei*[ti] AND thromb*[ti] 5362  #30 deep*[ti] AND vein*[ti] AND embol*[ti] 522  #31 deep*[ti] AND ven*[ti] AND embol*[ti] 31  #32 pulmonary thromb*[tiab] 4085  #33 pulmonary embol*[tiab] 34927  #34 lung thromb*[tiab] 51  #35 lung embol*[tiab] 396  #36 (pulmonar*[ti] OR lung*[ti]) AND (thromb*[ti] OR embol*[ti]) 22817  #37 DVT[tiab] 8904  #38 PE[tiab] 32675  #39 VTE[tiab] 8559  #40 #17 OR #18 OR #19 OR #20 OR #21 OR #22 OR #23 OR #24 OR #25 OR #26 OR #27 OR #28 OR #29 OR #30 OR #31 OR #32 OR #33 OR #34 OR #35 OR #36 OR #37 OR #38 OR #39 291953  #41 "Heparin"[Mesh] 61644  #42 "Heparin, Low-Molecular-Weight"[Mesh] 11445  #43 "Coumarins"[Mesh] 47190  #44 "Stockings, Compression"[Mesh] 1324  #45 "Intermittent Pneumatic Compression Devices"[Mesh] 584  #46 "Anticoagulants"[Mesh] 72793  #47 "Warfarin"[Mesh] 17592  #48 thromboprophyl*[tiab] 4108  #49 prophyla*[tiab] 147368  #50 heparin*[tiab] 81958  #51 nadroparin[tiab] OR fraxiparin[tiab] OR enoxaparin[tiab] OR clexane[tiab] OR lovenox[tiab] OR dalteparin[tiab] OR fragmin[tiab] OR ardeparin[tiab] OR normiflo[tiab] OR tinzaparin[tiab] OR logiparin[tiab] OR innohep[tiab] OR certoparin[tiab] OR sandoparin[tiab] OR reviparin[tiab] OR clivarin[tiab] OR danaproid[tiab] OR orgaran[tiab] OR anticoagulant*[tiab] OR Warfarin[tiab] OR Coumadin*[tiab] OR apo-warfarin[tiab] OR gen-warfarin[tiab] OR warfant[tiab] OR coumadin[tiab] OR aldocumar[tiab] OR tedicumar[tiab] OR acenocumarol[tiab] OR phenprocumon[tiab] OR vitamin K antagonist[tiab] OR VKA[tiab] OR fondaparinux[tiab] OR ximelagatran[tiab] OR dabigatran[tiab] OR apixaban[tiab] OR rivaroxaban[tiab] OR antiplatelet*[tiab] OR aspirin*[tiab] OR acetylsalicylic acid[tiab] OR acylpyrin[tiab] OR aloxiprimum[tiab] OR colfarit[tiab] OR dispril[tiab] OR easprin[tiab] OR ecotrin[tiab] OR endosprin[tiab] OR magnecyl[tiab] OR micristin[tiab] OR polopirin[tiab] OR polopiryna[tiab] OR solprin[tiab] OR solupsan[tiab] OR zorprin[tiab] OR acetysal[tiab] 136074  #52 stocking*[tiab] 4618  #53 bandage*[tiab] 4874  #54 (compression[tiab] OR pneumatic[tiab]) AND (device*[tiab] OR dress*[tiab]) 7476  #55 #41 OR #42 OR #43 OR #44 OR #45 OR #46 OR #47 OR #48 OR #49 OR #50 OR #51 OR #52 OR #53 OR #54 413723  #56 #40 AND #55 69164  #57 antithromb*[tiab] 31449  #58 thromboprophy*[tiab] 4108  #59 “Thromboembolism/prevention and control”[Majr] 8022  #60 #56 OR #57 OR #58 OR #59 94590  #61 #16 AND #60 649 |
| --- | --- |
| **EMBASE**  Ovid Embase<1980 to 2018 Week 13>  26/03/2018 | 1 exp Vein Thrombosis/ (112931)  2 exp Lung Embolism/ (80230)  3 exp venous thromboembolism/ (129461)  4 exp deep vein thrombosis/ (51486)  5 exp Thrombophlebitis/ (15042)  6 (thrombus* or thrombotic* or thrombolic* or thromboemboli* or thrombos* or thromboph*).ab,ti. (301251)  7 (deep* adj3 (vein* or ven*) adj5 (thromb* or embol*)).ab,ti. (35269)  8 (deep* adj3 (vein* or ven*) adj5 (thromb* or embol*)).ab,ti. (35269)  9 ((pulmonary or lung*) adj3 (thromb* or embol*)).ab,ti. (61326)  10 (DVT or PE or VTE).ab,ti. (72584)  11 1 or 2 or 3 or 4 or 5 or 6 or 7 or 8 or 9 or 10 (430715)  12 (thromboprophylaxis or prophylactic* or prophylaxis).ab,ti. (202369)  13 exp Heparin/ (129682)  14 exp Low Molecular Weight Heparin/ (55158)  15 expHeparinoid/ (1493)  16 exp compression garment/ (4369)  17 exp Compression Bandage/ (2076)  18 exp intermittent pneumatic compression device/ (959)  19 expVena Cava Filter/ (4733)  20 exp Anticoagulant Agent/ (581904)  21 expCoumarin Anticoagulant/ (90329)  22 expAntithrombocytic Agent/ (304722)  23 exp Acetylsalicylic Acid/ (184851)  24 Heparin*.ab,ti. (99917)  25 (Anticoagulant* or Warfarin or Coumadin* or apo-warfarin or gen-warfarin or warfant or Coumadin or aldocumar or tedicumar).ab,ti. (96866)  26 (Antiplatelet* or (platelet* adj3 aggregation adj3 inhibit*) or ((blood or platelet*) adj3 (antagonist* or antiaggrega*))).ab,ti. (48549)  27 (Aspirin* or acetylsalicylic acid or acylpyrin or aloxiprimum or colfarit or dispril or easprin or ecotrin or endosprin or magnecyl or micristin or polopirin or polopiryna or solprin or solupsan or zorprin or acetysal).ab,ti. (72331)  28 ((vena adj3 cava adj3 filter*) or (umbrella adj3 filter*)).ab,ti. (3345)  29 (Pentassacharide* or fondaparinux).ab,ti. (2575)  30 ((compression or impulse or pneumatic or elastic*) adj3 (device* or stocking* or hose* or dressing* or bandage*)).ab,ti. (7006)  31 12 or 13 or 14 or 15 or 16 or 17 or 18 or 19 or 20 or 21 or 22 or 23 or 24 or 25 or 26 or 27 or 28 or 29 or 30 (824624)  32 exp spine disease/ (193827)  33 spine/su [Surgery] (791)  34 exp spinal nerve/su [Surgery] (2437)  35 exp spine fusion/ (25058)  36 spine.ti,ab. (134011)  37 spinal.ti. (127265)  38 spinal stenos*.ti,ab. (5632)  39 spinal fusion.ti,ab. (6770)  40 spondylitis.ti,ab. (21175)  41 spondylosis.ti,ab. (3504)  42 scoliosis.ti,ab. (21922)  43 kyphosis.ti,ab. (9156)  44 kyphoscoliosis.ti,ab. (1791)  45 lordosis.ti,ab. (7004)  46 lumbar fusion.ti,ab. (2256)  47 32 or 33 or 34 or 35 or 36 or 37 or 38 or 39 or 40 or 41 or 42 or 43 or 44 or 45 or 46 (388292)  48 11 and 31 and 47 (1569) |
