# Supplementary material for: Thromboprophylaxis in elective spinal surgery: A protocol for systematic review
Source: Medicine (Baltimore). 2020 May 22;99(21):e20127. doi: 10.1097/MD.0000000000020127 (PMC7249943; doi:10.1097/MD.0000000000020127)
Supplement: Supplemental Digital Content [file medi-99-e20127-s002.docx]

**Appendix 2: Characteristics of included studies**

This appendix provides the general characteristics of included studies. For each primary study, we report:

- Study ID: Short name for identification of the study
- Design of the Study: As appraised by authors of this systematic review.
- Country: Country in which the study was carried on. If not stated, then country of the first author will be considered.
- Period: Referred to the period of time in which the study was conducted.
- Patients: Detailed description of inclusion and exclusion criteria for the participants of the primary study.
- Intervention/s: All the mechanical and/or pharmacological thromboprophylaxis interventions given to the participants.
- Outcome/s: Referred to all the outcomes reported in primary studies that wererelevant for this systematic review (DVT-PE-Bleeding). For this section, we did not consider outcomes that were not relevant for this systematic review (e.g. readmission, mortality).
- Follow up period: Referred to the follow up period that the authors of primary studies reported for assessing their outcomes.
- Main Results: describing the total of patients included, sex, age, and the cases of DVT-PE-Bleeding (if the study reported those outcomes). In case the study reported specific diagnosis, surgical level, surgical procedures or other relevant characteristics, this was reported too in the Main Results section.
- Comments: Describing the definition of DVT-PE-Bleeding, and any other relevant comment from the authors of this systematic review.

| **Study ID** | **Akeda 2014** |
| --- | --- |
| **Design of the study** | Prospective cohort study |
| **Country** | Japan |
| **Period** | 2006 – 2011 |
| **Patients** | Inclusion criteria: Patients who underwent spinal surgery  Exclusion criteria: Not reported |
| **Intervention/s** | Pneumatic sequential compression devices and standard compression stockings |
| **Outcome/s** | DVT, PE |
| **Follow up period** | Not clearly reported, but “ultrasonographic scans were performed preoperatively and repeated within 4 days after surgery” |
| **Main results** | \| Total patients included \| 209 \| \| --- \| --- \| \| Sex, female (cases) \| 88 \| \| Age (years) \| 64 + 17,1 \| \| Diagnosis (cases)   - Degenerative spinal diseases - Metastatic spinal tumor - Primary spinal tumor and spinal cord tumor - Spinal trauma - Other \| 147  21  7  10  24 \| \| Surgical procedure (cases)   - Lumbar decompression - Lumbar laminectomy and disectomy \|  \| \| DVT cases* \| 7 \| \| PE cases* \| 0 \|   *Excluding tumoral and traumatic diseases |
| **Comments** | ***For purposes of this systematic review, wecollected data only from de 147 patients( out of a total of 209 recruited) from the degenerative spinal diseases group***  Definition of DVT: “Results of ultrasonography were considered positive if a vein or venous segment was not fully compressible”  Definition of PE: Notreported |

| **Study ID** | **Al-Dujaili 2012** |
| --- | --- |
| **Design of the study** | Prospective cohort study |
| **Country** | United Arab Emirates |
| **Period** | Not reported |
| **Patients^1^** | Inclusion criteria: Patients who had spinal surgery performed  Exclusion criteria: Patients who underwent same-day procedures such as facet infiltration, percutaneous vertebroplasty, epidural injection, and medial branch thermo-coagulation |
| **Intervention/s** | Below-knee compressive elastic stockings + Enoxaparine 40 mg SC 12 to 13 hours postoperatively and continued daily until the patient was ambulating well and then was discharged.  Also, a sequential compressive device (SCD) was applied in special circumstances; perioperative, when anticipating prolonged operative time (more than 6 hours), and postoperatively in patients with severe neurologic impairment, paraplegia, or prolonged immobility. |
| **Outcome/s** | DVT, Bleeding |
| **Follow up period** | 6 months |
| **Main results** | \| Total patients included \| 158 \| \| --- \| --- \| \| Sex (% female) \| 39% \| \| Age (years) \| 43 (12-90) \| \| Surgical level (cases)   - Lumbar - Cervical \| 74%  26% \| \| DVT cases \| 1 \| \| Bleeding cases \| 3 \| |
| **Comments** | Definition of DVT: Clinical evaluation + routine ultrasound based imaging (second and third postoperative days), with or without D-dimer measurement  Definition of Bleeding: Spinal epidural hematoma  ***^1^ The study population consisted of a mixed population (thirteen patients underwent emergency spinal surgery, 10 for spinal trauma and 3 for acute cauda-equina syndrome)*** |

| **Study ID** | **Cox 2014** |
| --- | --- |
| **Design of the study** | Uncontrolled Before-After Study |
| **Country** | USA |
| **Period** | 2005 – 2010 |
| **Patients^1^** | Inclusion criteria: Patients > 18 years old, admitted for more than 24 hours, undergoing spine neurosurgery (in about 50% the primary diagnosis was degeneration or deformity)  Exclusion criteria: Not specified |
| **Intervention/s** | Group A (Pre-protocol group): Sequential compression devices (SCDs)  Group B (Post-protocol group): SCDs + Heparin (5000 U) SC 3 times daily, except those older than 75 years or weighing less than 50 kg, who receive dosing twice daily |
| **Outcome/s** | DVT, PE, Bleeding |
| **Follow up period** | Not reported |
| **Main results** | \|  \| Group A \| Group B \| \| --- \| --- \| --- \| \| Total patients included \| 941 \| 992 \| \| Sex (cases, % female) \| 427 (45,4%) \| 485 (48,9%) \| \| Age (years) \| 52,5 (18-99) \| 54,5 (18-89) \| \| Diagnosis   - Tumor - Vascular disease - Infection - Degeneration - Deformity - Trauma - Postop complication - Other \| 110 (10,9%)  25 (2,5%)  30 (3,0%)  484 (47,8%)  19 (1,9%)  189 (18,7%)  70 (6,9%)  85 (8,4%) \| 89 (8,3%)  41 (3,8%)  37 (3,4%)  457 (42,6%)  48 (4,5%)  194 (18,1%)  81 (7,5%)  127 (11,8%) \| \| Surgical procedures   - Spinal lesion - Decompression - Cervical fusion - Thoracolumbar fusion - Vertebroplasty - Other \| 120 (11,9%)  385 (38,0%)  326 (32,2%)  528 (52,2%)  5 (0,5%)  434 (42,9%) \| 104 (9,7%)  462 (43,0%)  314 (29,2%)  595 (55,4%)  9 (0,8%)  464 (43,2%) \| \| DVT cases \| 25 (2,7%) \| 10 (1,0%) \| \| PE cases \| 6 (0,6%) \| 5 (0,5%) \| \| Bleeding cases \| 6 (0,6%) \| 4 (0,4%) \| |
| **Comments** | ***^1^This study did not provide data of DVT and PE cases according to diagnosis subgroups, so, for purposes of this systematic review, we considered all the patients included (mixed population).***  Definition of DVT: Specified according to codification of the specific disease (ICD9)  Definition of PE: Specified according to codification of the specific disease (ICD9)  Definition of Bleeding: Postoperative hematoma in the surgical area requiring operative evacuation |

| **Study ID** | **Dearborn 1999** |
| --- | --- |
| **Design of the study** | Ambidirectional cohort study |
| **Country** | USA |
| **Period** | 1992 – 1994 |
| **Patients** | Inclusion criteria: Not clearly specified  Exclusion criteria: Not clearly specified |
| **Intervention/s** | Patients from both groups (Group A: Prospective group - Group B: Retrospective series) received thigh-length compression stockings and pneumatic compression leggings |
| **Outcome/s** | DVT, PE |
| **Follow up period** | Mean follow up (group A): 6 days (3-20 days for DVT, 3-58 days for PE) |
| **Main results** | \|  \| Group A \| Group B \| \| --- \| --- \| --- \| \| Total patients included \| 116 \| 318 \| \| Sex (cases, female) \| 82 \| 179 \| \| Age (years) \| 46 (16 – 78) \| 48 (15 – 80) \| \| Diagnosis   - Scoliosis - Spinal stenosis - Spondylolisthesis - Kyphosis - Other degenerative - Pseudarthrosis - Other \| 35  15  22  16  7  17  4 \| 71  38  44  31  18  41  24 \| \| Surgical procedures   - PSF - ASF - ASF/PSF - Hardware removal \| 62  3  49  2 \| 199  20  97  2 \| \| DVT cases \| 1 \| 1 \| \| PE cases \| 3 \| 7 \| |
| **Comments** | Definition of DVT: According to Duplex scans: Incompressibility of the echogenic lumen, blunted or absent Doppler waveform, absence of augmentation of flow proximal to an incompressible vein during compression of calf muscles, and absence of variation with respiration below the incompressible vein  Definition of PE: According to V/Q scans results  *These definitions were specified only for group A* |

| **Study ID** | **Du 2015** |
| --- | --- |
| **Design of the study** | Randomized controlled trial |
| **Country** | China |
| **Period** | 2009 – 2012 |
| **Patients** | Inclusion Criteria: Patients who underwent lumbar surgery, with one of the following risk factors for venous thromboembolism: Age > 60 years; body mass index > 39 kg/m2; thrombophilia, including conditions such as hypertension, diabetes, hyperlipidemia, cancer, and others; previous history of venous thrombosis; anterior or combined anterior–posterior procedure; and spinal trauma or spinal cord damage.  Exclusion Criteria: Oral anticoagulant therapy 3 months prior to the operation; vein thrombosis on preoperative B-ultrasound; preoperative urinalysis positive for red blood cells, fecal occult blood, skin purpura, or hematoma; active bleeding or high riskof bleeding; contraindication towards rivaroxaban and parnaparin or patients whose parnaparin dose needed to be adjusted. |
| **Intervention/s** | Group A: Rivaroxaban (oral) 10 mg 6 to 8 h after surgery, and once per day until the 14th day  Group B: Parnaparin (subcutaneous injections) 40 mg 6 to 8 h after surgery and once per day until the 14th day |
| **Outcome/s^1^** | DVT, PE, Bleeding |
| **Follow up period** | 14 days |
| **Main results** | \|  \| Group A \| Group B \| \| --- \| --- \| --- \| \| Total patients included \| 341 \| 324 \| \| Sex (F:M) \| Not reported \| Not reported \| \| Age (years) \| No specific data (“298 patients were older than 60 years of age”) \| \| \| DVT cases (total)   - Total DVT cases excluding patients with spinal trauma or spinal tumor \| 6  3 \| 10  5 \| \| PE cases \| 1 \| 2 \| \| Bleeding cases \| 21 \| 17 \| |
| **Comments** | ***^1^ For purposes of the meta-analysis, both groups were considered as one in the DVT outcome, because it was possible, in this case, to exclude the patients with spinal trauma or spinal tumor (there were 550 patients in total without these diseases). In the case of PE and Bleeding, with the available data it was impossible to exclude these patients, so we report the result for each group separately.***  Definition of DVT: Symptoms (leg swelling, lower extremity pain, Homans sign) + Doppler ultrasound, or Doppler ultrasound alone  Definition of PE: Symptoms (sudden-onset dyspnea, retrosternal pain, hemoptysis, cyanosis, syncope, profuse sweating, cold limbs, convulsions), + CT/angiography  Definition of Bleeding: Included fatal bleeding, bleeding in inflow critical organs, bleeding-induced reoperation, clinically significant bleeding outside the surgical site with a decrease of ≥20 g/L in hemoglobin level or the need to transfuse ≥2 units of whole blood or packed red blood cells, non-serious bleeding during the treatment and bleeding wound complications |

| **Study ID** | **Epstein 2005** |
| --- | --- |
| **Design of the study** | Prospective cohort study |
| **Country** | USA |
| **Period** | Not reported |
| **Patients** | Inclusion criteria: Patients undergoing single-level anterior corpectomy with fusion (ACF), and patients undergoing multilevel ACF (2–4 levels) with posterior fusion  Exclusion criteria: Not reported |
| **Intervention/s^1^** | Intermittent compression stockings for both groups (Group A: Single ACF – Group B: Multilevel ACF) |
| **Outcome/s** | DVT, PE |
| **Follow up period** | Mean follow-up: 2,5 years (2,0 – 3,2) for group A; 5,3 years (2 – 15) for group B |
| **Main results** | \|  \| Group A \| Group B \| \| --- \| --- \| --- \| \| Total patients included \| 100 \| 100 \| \| Sex (% female) \| 43% \| 35% \| \| Age (years) \| 46 \| 55 \| \| DVT cases \| 1 \| 7 \| \| PE cases \| 1 \| 2 \| |
| **Comments** | ***^1^For purposes of meta-analysis and considering that the intervention was the same for all the included patients, both groups were analyzed as one***  Definition of DVT and PE: “Doppler studies of the lower extremities were routinely obtained 2 days after surgery. If DVT was documented, patients underwent spiral CT angiography (CTA) to screen for attendant PE. Subsequent Dopplers, abdominal/pelvic/lower extremity computerized tomography (CT) scans, and spiral CTA were performed only when patients developed symptoms of DVT (e.g., leg swelling, pain) and/or PE (shortness of breath, pleuriticchest pain)” |

| **Study ID** | **Epstein 2006** |
| --- | --- |
| **Design of the study** | Prospective cohort study |
| **Country** | USA |
| **Period** | Not reported |
| **Patients** | Inclusion criteria: Patients undergoing multilevel lumbar laminectomies with instrumented fusions  Exclusion criteria: Not reported |
| **Intervention/s** | Pneumatic compression stockings |
| **Outcome/s** | DVT, PE |
| **Follow up period** | Doppler exams were only routinely performed 2 days following surgery. Not clearly specified if there was a longer follow-up period. |
| **Main results** | \| Total patients included \| 139 \| \| --- \| --- \| \| Sex (cases, female) \| 61 \| \| Age (years) \| 53 (25 – 75) \| \| Diagnosis   - Degenerative spondylolisthesis - Spondylolisthesis with lysis \| 102  37 \| \| DVT cases \| 4 \| \| PE cases \| 1 \| |
| **Comments** | Definition of DVT and PE: “All patients underwent routine Doppler sonographic screening for DVT 2 days postoperatively. Doppler studies were repeated only when patients became symptomatic with presumed DVT or PE. DVT was demonstrated utilizing Doppler ultrasound, whereas PE and DVT could both be confirmed/diagnosed on CT angiography of the chest, abdomen/pelvis, and lower extremities” |

| **Study ID** | **Fawi 2017** |
| --- | --- |
| **Design of the study** | Retrospective cohort study |
| **Country** | UK |
| **Period** | 2007 – 2012 |
| **Patients** | Inclusion criteria: Patients who had undergone elective surgery of the thoracic and/or lumbar spine  Exclusion criteria:Previously documented venous thromboembolism (VTE), trauma patients, patients having surgery for tumor or infection, those with a known bleeding disorder, patients who had been treated with anticoagulant (e.g. for valve replacements), and patients < 16 years of age |
| **Intervention/s** | Group A: Below-knee TED anti-embolic stockings on admission and until four weeks postoperatively (unless the patient was deemed high risk of VTE complications at the pre-operative assessment, in which case chemoprophylaxis was also given)  Group B: Same as group A + Subcutaneous enoxaparin-a LMWH, 40 mg injection six hours post-operatively and administered for the duration of the hospitalization. |
| **Outcome/s** | DVT, PE, Bleeding |
| **Follow up period** | 90 days |
| **Main results** | \|  \| Group A \| Group B \| \| --- \| --- \| --- \| \| Total patients included* \| 1677** \| 689** \| \| Sex (cases, female) \| 942 \| 353 \| \| Age (years) \| 46,8 (16 – 94) \| \| \| Surgical procedure   - Decompression/disectomy - Decompression and interspinous spacer - Interbody fusion - Postero-lateral lumbar fusion - Extended thoracolumbar fusion - Removal of metal-work \| 510  156  472  392  126  22 \| 135  173  293  74  0  13 \| \| DVT cases \| 1 \| 0 \| \| PE cases \| 9 \| 0 \| \| Bleeding cases \| 0 \| 0 \|   * 2366 procedures in 2181 patients.  **In the text, the study specifies that “There were 1677 procedures in the control group and 689 in the LMWH group”. Nevertheless, the sum of their respective subgroups (according to table 1) are 1678 and 688. In another table (table 4) the values are, again, 1677 and 689. So we used this last data for our meta-analysis. |
| **Comments** | Definition of DVT and PE: According to symptoms and imaging (“If VTE was suspected, the patient was investigated with a duplex scan of the lower limb veins for potential DVT or a CT pulmonary angiogram/ ventilation-perfusion scan for symptomatic pulmonary emboli”)  Definition of Bleeding: Epidural hematoma. |

| **Study ID** | **Ferree 1993** |
| --- | --- |
| **Design of the study** | Prospective cohort study |
| **Country** | USA |
| **Period** | Not reported |
| **Patients** | Inclusion criteria:Consecutive patients undergoing posterior lumbar spinal surgery (84 patients underwent either laminectomy or laminotomy procedures while 101 underwent posterior spinal fusion procedures with or without laminectomy or laminotomy)  Exclusion criteria:Not reported |
| **Intervention/s** | Group A: Elastic compression stockings  Group B: Intermittent pneumatic compression (begun intraoperatively) combined with eastic compression stockings  *“The patients were selected from groups of two surgeons, both of whom were using different methods of DVT prophylaxis”.* |
| **Outcome/s** | **DVT, PE** |
| **Follow up period** | 2 – 7 days |
| **Main results** | \|  \| Group A \| Group B \| \| --- \| --- \| --- \| \| Total patients included \| 74 \| 111 \| \| Sex (cases, female) \|  \|  \| \| Age (years) \| 43 \| 50 \| \| Surgical procedures   - Laminectomy or laminotomy - Spinal fusion (with or without laminectomy or laminotomy) \| 40  34 \| 44  67 \| \| DVT cases \| 4 \| 0 \| \| PE cases \| 0 \| 0 \| |
| **Comments** | Definition of DVT: According to results of postoperative duplex ultrasound  Definition of PE: Not reported |

| **Study ID** | **Ferree 1994** |
| --- | --- |
| **Design of the study** | Prospective cohort study |
| **Country** | USA |
| **Period** | Not reported |
| **Patients** | Inclusion criteria: Patients undergoing lumbar laminotomy or laminectomy  Exclusion criteria: Not reported |
| **Intervention/s** | Compression stockings |
| **Outcome/s** | DVT, PE |
| **Follow up period** | Not clearly specified |
| **Main results** | \| Total patients included \| 60 \| \| --- \| --- \| \| Sex (cases, female) \| 26 \| \| Age (years) \| 46 (14 – 80) \| \| Diagnosis and procedures   - Laminotomy for herniated lumbar disc - Laminectomy spinal stenosis \| 51  9 \| \| DVT cases \| 3 \| \| PE cases \| 0 \| |
| **Comments** | Definition of DVT: According to results of compression ultrasonography  Definition of PE: According to symptoms (not specified) |

| **Study ID** | **Gerlach 2003** |
| --- | --- |
| **Design of the study** | Retrospective cohort study |
| **Country** | Germany |
| **Period** | 1999 – 2002 |
| **Patients** | Inclusion criteria: Patients undergoing spinal surgery  Exclusion criteria: Not reported |
| **Intervention/s** | Early postoperative daily subcutaneous administration of 0.3 ml of nadroparin calcium (Fraxiparin) (2850 IU anti-Xa) + intra- and post-operative compression stockings |
| **Outcome/s** | DVT, PE, Bleeding |
| **Follow up period** | Not reported |
| **Main results** | \| Total patients included \| 1954* \| \| --- \| --- \| \| Sex (cases, female) \| Not reported \| \| Age (years) \| Not reported \| \| Level of procedure   - Cervical - Thoracic - Lumbar \| 503  152  1299 \| \| Surgical Procedures (Cervical):   - Cervical disc herniation and/or spinal stenosis (ventral fusion) - Miscellaneous tumor surgery - Cervical abscess and empyema - C1/2 posterior stabilization - Stabilization of cervical spine fractures - Anterior screw fixation of dens fractures - Spontaneous intraspinal hematoma \| 390  37  21  23  23  7  2 \| \| Surgical Procedures (Thoracic):   - Decompression of spinal cord (metastasis) - Miscellaneous tumor surgery - Meningioma - Thoracic disc herniation - Spontaneous intraspinal hematoma - Abscess and empyema - Decompression of syrinx - Traumatic fractures - Occlusion of arterio-venous fistulae \| 48  37  16  13  12  11  5  5  5 \| \| Surgical Procedures (Lumbar):   - Lumbar disc herniation and/or spinal stenosis - Miscellaneous tumor surgery - Posterior and/or anterior stabilization procedures - Abscess and empyema \| 1215  44  20  20 \| \| DVT cases \| 1 \| \| PE cases \| 0 \| \| Bleeding cases \| 13 \|   **Reported as “procedures”, not “patients”.* |
| **Comments** | Definition of DVT and PE: “Surveillance of VTE was done clinically. When patients presented with clinical criteria of a DVT or pulmonary embolism (PE; i.e,. pain and swelling of the leg, sudden onset of respiratory difficulties without a previous history of dyspnea), a venography was performed to confirm clinical diagnosis. However, no routine venography or duplex ultrasound was performed for DVT exclusion.”  Definition of Bleeding: “Major postoperative hemorrhage was defined as a hemorrhage associated with a mass effect on postoperative spinal MRI and/or neurological deterioration, as well as a large-wound hematoma with intractable pain. All patients with postoperative hematoma, who required surgical treatment, were retrieved from the operative database and analyzed retrospectively.” |

| **Study ID** | **Gruber 1984** |
| --- | --- |
| **Design of the study** | Randomized controlled trial |
| **Country** | Switzerland |
| **Period** | Not stated |
| **Patients** | Inclusion criteria: Adults undergoing lumbar disc operations  Exclusion criteria: Recurrent disease, epidural compression by metastases and patients with abnormal preoperative clotting tests |
| **Intervention/s^1^** | Group A: Miniheparindihydroergotamine (2500 IU twice daily, first dose given 2 h preoperatively)  Group B: Placebo |
| **Outcome/s** | DVT, PE, Bleeding |
| **Follow up period** | Not stated |
| **Main results** | \|  \| Group A \| Group B \| \| --- \| --- \| --- \| \| Total patients included \| 25 \| 25 \| \| Sex (F:M) \| 9:12 \| 7:13 \| \| Age (years) \| 47,4 + 13 \| 44,5 + 9,7 \| \| DVT cases \| 1 \| 0 \| \| PE cases \| 0 \| 0 \| \| Bleeding cases \| 6 \| 7 \|   ** Study does not report sex for patients included that showed preoperative changes in the clotting mechanism* |
| **Comments** | ***^1^ For purposes of this systematic review, only data from group A was considered, as this was the only cohort to report incidence with one of the interventions of interest.***  Definition of DVT: Not clearly defined, but it is stated that “If there was clinical suspicion of deep vein thrombosis, a phlebogram, plethysmography, Doppler ultrasound or an I^125^ fibrinogen test was performed”.  Definition of PE: Not clearly defined, but it is stated that “Patients suspected of having a pulmonary embolism received a chest X-ray, ECG, perfusion ventilation scintigram or a pulmonary angiogram”.  Definition of Bleeding: Not clearly defined, but it is stated that “At the end of each operation, the surgeon was asked if there had been increased bleeding”. The number describes increased intraoperative bleeding + deep wound hematoma. |

| **Study ID** | **Guo 2017** |
| --- | --- |
| **Design of the study** | Retrospective cohort study |
| **Country** | China |
| **Period** | 2013 – 2016 |
| **Patients** | Inclusion criteria: Patients who underwent lumbar surgery and needed spinal decompression due to trauma and degenerative diseases.  Exclusion criteria: Patients with DVT before surgery, patents using anticoagulants such as aspirin and warfarin for other diseases, patients younger than 18 years, patients with a history of hepatic impairment, renal/liver insufficiency, acute infections, active tuberculosis and malignant tumors. |
| **Intervention/s^1^** | Group A: Thigh-high compression stockings with a sequential compression device + Argabotran, 10 mg within 2 h bid with a micro-pump, starting 6-8 hours after surgery, for 7-14 days until the patient was able to ambulate independently  Group B: Thigh-high compression stockings with a sequential compression device + Low molecular weight heparin, subcutaneous injection of 2125 U 6 hours after surgery followed by 4250 U qd, for 7-14 days until the patient was able to ambulate independently |
| **Outcome/s** | DVT, PE, Bleeding |
| **Follow up period** | 4 weeks |
| **Main results^2^** | \|  \| Group A \| Group B \| \| --- \| --- \| --- \| \| Total patients included \| 274 \| 282 \| \| Sex (cases, female) \| 154 \| 150 \| \| Age (years) \| 66,3 + 6,8 \| 66,8 + 7,1 \| \| Type of surgery   - Fracture reduction decompression intrafixation - Intervertebral foramen approach vertebral interbody fusion (TLIF) - Posterior vertebral interbody fusion (PLIF) - Overhaulin operation \| 62  108  89  15 \| 76  98  98  10 \| \| DVT cases   - Total cases excluding fractures \| 3*  2 \| 4**  3 \| \| PE cases \| 0 \| 0 \| \| Bleeding cases \| 1*** \| 4**** \|   *Of these, 1 was symptomatic and 2 were asymptomatic  ** All these cases were asymptomatic  *** Incisional bleeding  **** 1 large subcutaneous ecchymosis, 2 incisional bleedings, 1 epidural hematoma |
| **Comments** | Definition of DVT: Definite diagnosis of DVT was made by Duplex ultrasound. Doppler ultrasound on the bilateral deep veins of the lower extremities was performed before surgery, at 7 and 14 days after surgery, and at 4weeks after ending of the treatment. Patients were monitored daily for symptoms and signs of DVT  Definition of PE: Patients were monitored daily for symptoms and signs of PE. Computed tomography was also used to evaluate suspected PEevents after surgery.  Definition of Bleeding: Severe bleeding was defined as fatal bleeding, bleeding in inflow critical organs, such as the posterior peritoneum, intracranium, intraocular and intraspinal canal, bleeding-induced reoperation, or clinically significant bleeding outside the surgical site with a >20 g/L decrease in hemoglobin (Hb) level or the need for >2 units of whole blood or packed red blood cell transfusion. Non-severe bleeding contained other bleeding events that were not evaluated as severe bleeding, such as skin bruising, fecal occult blood, gastrointestinal bleeding and urine erythrocytes.  ***^1^For purposes of analyses, both groups were treated as one, as they both provided chemical and mechanical thromboprophylaxis.***  ***^2^For the DVT and PE outcomes, we considered 5 cases and 0 cases respectively, because we excluded patients with fracture. Therefore, the total considered for these outcomes was 418 patients (patients with fracture excluded). In the case of bleeding, it was not possible to separate the data, so we considered all patients.*** |

| **Study ID** | **Hamidi 2015** |
| --- | --- |
| **Design of the study** | Randomized controlled trial |
| **Country** | Iran |
| **Period** | 2011 |
| **Patients** | Inclusion criteria: Adults between 18 to 75 years old who underwent an elective instrumental spinal surgery  Exclusion criteria: History of recent active or major bleeding, hemophilia, low platelet count (<100000/mL) or platelet dysfunction, LMWH hypersensitivity, esophageal varices, hepatic impairment, renal insufficiency, uncontrolled hypertension, anemia (Hb<13 g/dLfor men, Hb<12 g/dL for women), acute infective endocarditis, active tuberculosis, history of chemotherapy prior to surgery, history of IV drug use, presence of spinal cord injury and history of spinal tumors. |
| **Intervention/s** | Group A: Enoxaparin 40 mg/day, within 12 hours before the surgery + Compression stockings after surgery  Group B: Compression stockings after surgery |
| **Outcome/s** | DVT, PE, Bleeding |
| **Follow up period** | 8 months |
| **Main results** | \|  \| Group A \| Group B \| \| --- \| --- \| --- \| \| Total patients included \| 40 \| 49 \| \| Sex (F:M) \| 21:19 \| 22:27 \| \| Age (years) \| 53,4 + 15,7 \| 50,1 + 13,8 \| \| Diagnosis (%)   - Herniated disc - Spinal stenosis - Spondylolisthesis - Scoliosis - Kyphosis - Post traumatic deformities \| 17 (42,5%)  19 (47,5%)  9 (22,5%)  2 (5,0%)  1 (2,5%)  0 (0%) \| 24 (48,9%)  27 (55,1%)  10 (20,4%)  4 (8,1%)  1 (2,0%)  1 (2,0%) \| \| Surgical level, cases (%)   - Cervical - Thoracic - Lumbar - Multilevel \| 8 (20,0%)  1 (2,5%)  29 (72,5%)  2 (5,0%) \| 12 (24,4%)  1 (2,0%)  35 (71,4%)  1 (2,0%) \| \| DVT cases \| 0 \| 2 \| \| PE cases \| 1 \| 0 \| \| Bleeding cases \| 0 \| 1 \| |
| **Comments** | Definition of DVT: Confirmed by compression Doppler ultrasonography  Definition of PE: Confirmed by imaging during hospitalization, or by imaging or autopsy within the follow-up period  Definition of Bleeding: Symptomatic epidural/wound hematoma |

| **Study ID** | **Hohl 2015** |
| --- | --- |
| **Design of the study** | Retrospective cohort study |
| **Country** | USA |
| **Period** | 2000 – 2009 |
| **Patients** | Inclusion criteria: Elective major thoracolumbar degenerative spine surgery, defined as multilevel laminectomies or laminotomies, and all fusions (instrumented and noninstrumented). Degenerative disorders: Spondylosis, spondylolisthesis, and degenerative scoliosis. Anterior, posterior, and combined approaches.  Exclusion criteria: Herniated nucleus pulposis treated with a single-level laminectomy or laminotomy (although included if they had a fusion). Neoplasm, trauma, infection, and idiopathic deformity |
| **Intervention/s** | Thigh-high TED hose and pneumatic sequential compression devices intraoperatively and postoperatively |
| **Outcome/s** | DVT, PE, Bleeding |
| **Follow up period** | 6 months |
| **Main results** | \| Total patients included \| 5766 \| \| --- \| --- \| \| Sex (cases, female) \| 3057 (53%) \| \| Age (years) \| 60,3 \| \| Revision surgery  Surgery with instrumentation \| 34.7%  62.6% \| \| DVT cases (symptomatic) \| 38 \| \| PE cases (symptomatic) \| 51  (one patient died) \| \| Bleeding cases \| 0 \| |
| **Comments** | Definition en DVT: “DVT was identified by crossmatching the spine attendings’ names with all records from the peripheral vascular laboratory. The records were searched for positive DVTs as diagnosed by lower extremity venous duplex scans. Patients were considered to have a DVT related to surgery if the DVT occurred within 6 months of the index procedure”  Definition of PE: “PE was identified by cross-matching the spine attendings’ names with the records for symptomatic PEs as diagnosed by spiral chest CT scans, nuclear scintigraphic ventilation-perfusion, and pulmonary angiography.”  Definition of Bleeding: Symptomatic epidural hematoma |

| **Study ID^1^** | **McLynn 2017 – NSQIP (National) Database Cohort** | **McLynn 2017 – Academic Center Database Cohort**  **(subgroup from NSQIP Database)** |
| --- | --- | --- |
| **Design of the study** | Retrospective cohort study | Retrospective cohort study |
| **Country** | USA | USA |
| **Period** | 2005 – 2014 | 2013 – 2016 |
| **Patients** | Inclusion criteria: Patients undergoing elective spine surgery.  Exclusion criteria: Patients were excluded for cases marked emergency or non-elective, fusion of seven or more levels (to exclude deformity cases with increased risks), missing data, and primary International Classification of Diseases diagnosis codes indicating trauma, tumor, or infection. | Inclusion criteria: Elective spine surgery patients recorded for NSQIP at a single large academic medical center.  Exclusion criteria: Same as National Database cohort |
| **Intervention/s** | Not reported | Sequential compression devices   - 56,1% of patients also received pharmacologic prophylaxis |
| **Outcome/s** | VTE (DVT+PE) | VTE (DVT+PE), Bleeding |
| **Follow up period** | 30 days | 30 days |
| **Main results** | \| Total patients included \| 109609 \| \| --- \| --- \| \| Sex (cases, female) \| 52628 \| \| Age (years) \| 56,4 + 14 \| \| DVT cases \| 672 \| \| PE cases \| | \| Total patients included   - With pharmacologic prophylaxis \| 2855  1603 \| \| --- \| --- \| \| Sex (cases, female) \| 1364 \| \| Age (years) \| 56,0 + 14,7 \| \| DVT cases \| 35 \| \| PE cases \| \| Bleeding cases (total)   - Received pharmacologic thromboprophylaxis - Did not received pharmacologic thromboprophylaxis \| 11  10  1 \| |
| Comments | Definition of DVT and PE: National database specified that all thromboembolic events were confirmed with imaging. | Definition of DVT and PE: Institutional data included only symptomatic VTEs, confirmed with venous duplex ultrasonography (in the case of DVT) or computed tomography angiogram or ventilation-perfusion scan (in the case of PE).  Definition of Bleeding: Postoperative bleeding or hematoma requiring a return to the operating room. |
|  | ***^1^For purposes of this systematic review, we only considered the cohort from the Academic Center database, because the intervention in the National database cohort was not clear. The study did not provide data of DVT and PE separately, so we did not include this data into meta-analysis.*** | |

| **Study ID** | **Moayer 2016** |
| --- | --- |
| **Design of the study** | Retrospective cohort study |
| **Country** | Iran |
| **Period** | 2004 – 2005 |
| **Patients** | Inclusion criteria: Patients who were scheduled for elective degenerative spine surgeries and received prophylactic dosages of deltaparin postoperatively  Exclusion criteria: Patients with contraindications of anticoagulation therapy including intracranial bleeding, severe active bleeding, recent brain, eye, or spinal cord surgery, pregnancy, malignant hypertension, those with recent major surgery, recent cerebrovascular accident, severe thrombocytopenia , or decreased renal function (serum creatinine>2mg/dL) |
| **Intervention/s** | Daily subcutaneous injection of 5000 unit Dalteparin (FRAGMIN), minimun for 3 days. |
| **Outcome/s** | DVT, PE, Bleeding |
| **Follow up period** | 90 days |
| **Main results** | \| Total patients included \| 120 \| \| --- \| --- \| \| Sex (cases, female) \| 66 (55%) \| \| Age (years) \| 44,8 + 12,6 \| \| Level of surgery   - Cervical - Thoracolumbar - Lumbar \| 23 (19%)  4 (3,5%)  93 (77,5%) \| \| Surgical procedure   - Lumbar discectomy - Lumbar laminectomy - Lumbar fixation - ACDF - Cervical laminectomy - Instrument removal - Vertebroplasty \| 31 (25,9%)  19 (14,8%)  40 (33,4%)  22 (18,3%)  1 (0,8%)  6 (5%)  1 (0,8%) \| \| DVT cases \| 1 \| \| PE cases \| 0 \| \| Bleeding cases \| 0 \| |
| **Comments** | Definition of DVT and PE: “Patients with clinically suspected DVT, underwent color Doppler sonography and those with clinical features suggestive of PE underwent chest perfusion scan.”  Definition of bleeding: Not reported |

| **Study ID** | **Nelson 1996** |
| --- | --- |
| **Design of the study** | Randomized controlled trial |
| **Country** | USA |
| **Period** | Not reported |
| **Patients** | Inclusion criteria: Patients undergoing posterolateral lumbar spinal fusion with pedicular fixation done in the knee-chest position  Exclusion criteria: Patients with neoplasm, trauma, scoliosis, or history of DVT. |
| **Intervention/s** | Group A: Thigh-high antiembolic compression stockings + aspirin 600 mg twice daily (all until discharge).  Group B: Thigh-high antiembolic compression stockings and pneumatic compression stockings + aspirin 600 mg twice daily (all until discharge). |
| **Outcome/s** | DVT, PE |
| **Follow up period** | Not clearly reported |
| **Main results** | \|  \| Group A \| Group B \| \| --- \| --- \| --- \| \| Total patients included \| 60 \| 57 \| \| Sex (cases, female) \| 32 \| 29 \| \| Age (years) \| 51 (20 – 69) \| 54 (20 – 82) \| \| Diagnosis   - Spondylolisthesis - Degenerative disk disease \| 15  45 \| 16  41 \| \| DVT cases \| 0 \| 0 \| \| PE cases \| 0 \| 0 \| |
| **Comments** | Definition of DVT: According to symptoms and ultrasonography ( “Continuous flow Doppler imaging, followed by duplex scanning, was done 3 to 6 days postoperatively”).  Definition of PE: Not provided. |

| **Study ID** | **Nicol 2009** |
| --- | --- |
| **Design of the study** | Retrospective cohort study |
| **Country** | UK |
| **Period** | 1985 – 2003 |
| **Patients** | Inclusion criteria: Patients who underwent lumbar spinal surgery  Exclusion criteria: Not reported |
| **Intervention/s^1^** | Group A (Cohort 1985 – 1994): No thromboprofilaxis  Group B (Cohort 1996 – 2003): Aspirin 150 mg daily from the first postoperative day, unless specifically contraindicated (in whichcase they were given low molecular weight heparin) + intermittent calf compression intraoperatively and compression stockings post operatively |
| **Outcome/s** | DVT, PE |
| **Follow up period** | 3 months |
| **Main results** | \|  \| Group A \| Group B \| \| --- \| --- \| --- \| \| Total patients included \| 697 \| 414 \| \| Sex (cases, female) \| Not reported \| \| \| Age (years) \| Not reported \| \| \| Surgical procedure   - Non-fusion - Fusion \| 554  143 \| 272  142 \| \| DVT cases \| 2 \| 1 \| \| PE cases \| 0 \| 0 \| |
| **Comments** | ***^1^ Although some patients in Group A received some form of mechanical prophylaxis, the way results are reported don’t allow to extract data specifically for these patients. Accordingly, for purposes of this systematic review, we only considered data corresponding to Group B (mixed thromboprophylaxis)***  Definition of DVT: Not reported, but patients were diagnosed by venography and duplex/doppler ultrasound scan  Definition of PE: Not reported |

| **Study ID** | **Pateder 2008** |
| --- | --- |
| **Design of the study** | Retrospective cohort study |
| **Country** | USA |
| **Period** | 1992 – 2000 |
| **Patients** | Inclusion criteria: Adults undergoing reconstructive spinal fusion procedures  Exclusion criteria: Not reported |
| **Intervention/s** | Thigh-length elastic antiembolism stockings + Sequential compression stockings (before the procedure and until full ambulation was regained) + Warfarin or low-molecular weight heparin*  *Patients received prophylactic warfarin from 1992 to 1999, starting on postoperative day 1. In the year 2000, low-molecular weight heparin became more readily available at the institution and patients were treated with low-molecular weight heparin or warfarin. |
| **Outcome/s** | DVT, PE, Bleeding |
| **Follow up period** | Minimum 3 years (average: 7 years) |
| **Main results** | \| Total patients included \| 407** \| \| --- \| --- \| \| Sex (cases, female) \| 340 \| \| Age (years) \| 48 (20 – 86) \| \| Type of surgery   - Primary spinal fusion surgery - Revision spinal fusion surgery \| 146  261 \| \| Diagnosis   - Scoliosis - Kyphosis - Scoliosis with pseudarthrosis - Kyphosis with pseudarthrosis \| 211  65  89  42 \| \| Level(s) of procedurs   - Less than 5 levels - 5-10 levels - More than 10 levels \| 108  192  107 \| \| DVT cases \| 6 \| \| PE cases \| 10 \| \| Bleeding cases \| 3 \|   **In total the study reported 407 surgical procedures in 361 adult patients. For purposes of analysis, 407 was taken as denominator for the incidence of DVT-PE-Bleeding |
| **Comments** | Definition of DVT: Symptoms + compatible duplex ultrasound  Definition of PE: “Patients did not undergo screening examinations, and diagnostic studies were performed only if there was a clinical suspicion of PE. Diagnoses were made by ventilation/perfusion scan or spiral computed tomography.”  Definition of Bleeding: Epidural hematomas requiring decompression and wound hematomas |

| **Study ID** | **Piasecki 2008** |
| --- | --- |
| **Design of the study** | Prospective cohort study |
| **Country** | USA |
| **Period** | 2002 – 2006 |
| **Patients** | Inclusion criteria: Patients undergoing combined anterior/posterior spinal reconstructions by 2 surgeons.  Exclusion criteria: Patients with a history of thromboembolic disease or any known hypercoagulability or contraindication to MRI were excluded. |
| **Intervention/s** | Foot pumps |
| **Outcome/s** | DVT, PE |
| **Follow up period** |  |
| **Main results** | \| Total patients included \| 66 \| \| --- \| --- \| \| Sex (cases, female) \| 57 \| \| Age (years) \| 52,7 + 9,6 \| \| Diagnosis   - Scoliosis - Revision surgery \| 40  26 \| \| DVT cases \| 6 \| \| PE cases \| 5 \| |
| **Comments** | Definition of DVT: “Surveillance bilateral lower extremity Doppler ultrasound and magnetic resonance venography studies were completed as close to postoperative day 7 as clinically feasible to evaluate the presence or absence of lower extremity and pelvic DVT, respectively.”  Definition of PE: “Clinical suspicion of PE, if present, prompted further evaluation with a contrast enhanced, spiral CT scan”. |

| **Study ID** | **Rokito 1996** |
| --- | --- |
| **Design of the study** | Randomized controlled trial |
| **Country** | USA |
| **Period** | 1993 – 1994 |
| **Patients** | Inclusion criteria: Patients undergoing major reconstructive spinal procedures, defined as those involving anterior and/or posterior spinal fusions and/or decompressions.  Exclusion criteria: Posterior spinal decompressions of only one level and one level lumbar discectomies. History of one or more of the following: Thrombophlebitis, pulmonary embolism, present treatment with anticoagulants, bleeding disorder, and peptic ulcer disease. |
| **Intervention/s^1^** | Group A: Bilateral thigh-high thrombosis embolic deterrent (TED) compression stockings  Group B: TED stockings and thigh-length cuffs that provided sequential pneumatic compression to the calf and thigh  Group C: TED stockings and low dose Coumadin |
| **Outcome/s** | DVT, PE, Bleeding |
| **Follow up period** | 1 year |
| **Main results** | \|  \| Group A \| Group B \| Group C \| \| --- \| --- \| --- \| --- \| \| Total patients included \| 42 \| 33 \| 35 \| \| Sex (% female) \| 60 \| 61 \| 60 \| \| Age (years) \| 46 (18-78) \| 45 (18-77) \| 44 (22-70) \| \| Diagnosis (%)   - Lumbar disorder - Scoliosis - Cervical disorder - Thoracic disorder - Spinal trauma \| 71  19  10  0  0 \| 88  6  0  0  6 \| 77  11  9  3  0 \| \| Surgical level (cases)   - Cervical - Thoracic - Lumbar - Scoliosis \| 4  0  30  8 \| 0  0  31  2 \| .  3  1  27  4 \| \| DVT cases \| 0 \| 0 \| 0 \| \| PE cases \| 0 \| 0 \| 0 \| \| Bleeding cases \| 0 \| 0 \| 2 \| |
| **Comments** | ***^1^ For purposes of this systematic review, data from groups A and B were considered as mechanical prophylaxis. Group C was considered as mixed thromboprophylaxis***  ***.***Definition of DVT: Duplex Doppler studies were performed in all patients between fifth and seventh postoperative day, or earlier when clinically indicated. Criteria of DVT well defined. If it was indeterminate or positive, venography was performed  Definition of PE: Not clearly defined. Apparently by symptoms  Definition of Bleeding: Not clearly defined, but events were adjudicated because “the postoperative (blood) losses were significantly greater requiring acute patient care” |

| **Study ID** | **Schizas 2008** |
| --- | --- |
| **Design of the study** | Prospective cohort study |
| **Country** | Switzerland |
| **Period** | Not reported |
| **Patients** | Inclusion criteria: Patients undergoing spinal surgery  Exclusion criteria: Patients undergoing procedures under local anaesthetic such as discographies and facet joint injections |
| **Intervention/s** | Compressive stockings + Enoxoparin starting from the eighth postoperative hour, once daily, and discontinued upon discharge (20 mg  during the first 3 postoperative days and 40 mg thereafter) |
| **Outcome/s** | PE, Bleeding |
| **Follow up period** | Minimum 12 months |
| **Main results** | \| Total patients included \| 270 \| \| --- \| --- \| \| Sex \| Not reported \| \| Age (years) \| 44,2 (15,6 – 92,4) \| \| Level of procedure   - Cervical - Lumbar - Thoracic or thoracolumbar \| 31  198  41 \| \| Diagnosis   - Spinal stenosis - Acute fractures or posttraumatic deformities - Degenerative disc disease - Herniated lumbas discs - Isthmic spondylolisthesis - Degenerative scoliosis with stenosis - Tumours - Infections - Rheumatoid arthritis \| 77  57  37  26  27  23  8  8  7 \| \| PE cases \| 6 \| \| Bleeding cases \| 2 \| |
| **Comments** | Definition of PE: Diagnosed with spiral chest computed tomography, only when clinical suspicion of PE was present  Definition of Bleeding: Postoperative hematomas requiring surgical evacuation |

| **Study ID** | **Smith 1994** |
| --- | --- |
| **Design of the study** | Prospective cohort study |
| **Country** | USA |
| **Period** | 1991 – 1992 |
| **Patients** | Inclusion criteria: Patients undergoing reconstructive operations of the spine, hospitalized for 4 or more days.  Exclusion criteria: Not reported |
| **Intervention/s** | Thigh-high compressive stockings (until patient was discharged from the hospital) + sequential pneumatic compression (until patient was able to walk) |
| **Outcome/s** | DVT, PE |
| **Follow up period** | 4 months minimum |
| **Main results** | \| Total patients included   - Without infection or spinal trauma \| 317  280 \| \| --- \| --- \| \| Sex \| Not reported \| \| Age (years) \| 39,2 \| \| Diagnosis   - Cervical lesion - Infection - Lumbar lesion - Scoliosis - Spinal trauma - Spondylolisthesis - Thoracic lesion \| 32  3  122  77  34  31  18 \| \| Type of operation   - Anterior - Posterior - Combined \| 32  132  153 \| \| DVT cases   - In patients without infection or spinal trauma \| 2  2 \| \| PE cases   - In patients without infection or spinal trauma \| 1  0 \| |
| **Comments** | Definition of DVT: Patients were observed daily for signs or symptoms of deep venous thrombosis. Duplex ultrasonography was used to screen for the presence of asymptomatic thrombosis in a random subset of 126 patients, on the fourth, fifth or sixth postoperative day. Diagnostic criteria for DVT included lack of compressibility of the venous lumen, filling of the lumen with echoes, blunter or absent Doppler waveform, a lack of augmentation of venous flow proximal to an incompressible vein during compression of the muscles of the calf, and a lack of effect of respiration in the Doppler waveform at and distal to the incompressible vein. If abnormal waveforms were the only findings, ascending venography was performed in order to rule out DVT.  Definition of PE: Not clearly defined. One fatal PE was diagnosed by autopsy in this study. |

| **Study ID** | **Strom 2013** |
| --- | --- |
| **Design of the study** | Retrospective cohort study |
| **Country** | USA |
| **Period** | 2007 – 2011 |
| **Patients** | Inclusion criteria: Patients undergoing decompressive laminectomy  Exclusion criteria: Patients with single-level decompression without fusion, non-degenerative disease (traumatic, infectious or neoplastic) |
| **Intervention/s** | Knee-high sequential compression devices on arrival to the operating room + intermittent pneumatic compression until discharge + daily prophylactic enoxaparin (40 mg for normal renal function, 30 mg for creatinine clearance of < 30 mL/min). |
| **Outcome/s** | DVT, PE, Bleeding |
| **Follow up period** | 30 days |
| **Main results** | \| Total patients included \| 367 \| \| --- \| --- \| \| Sex (cases, female) \| 163 \| \| Age (years) \| 64 \| \| Level of surgery   - Cervical - Lumbar \| 126  241 \| \| DVT cases \| 10 \| \| PE cases \| 4 \| \| Bleeding cases \| 0 \| |
| **Comments** | Definition of DVT: According to ultrasonography(“Lower extremity ultrasonography was performed on patients with a sign or symptom of DVT and those not mobilized by post-operative day 3”),  Definition of PE: According to chest computed tomography (CT) (“Chest CT with contrast was performed for patients with dyspnea, chest pain, desaturation, tachypnea, or tachycardia in whom PE could not be ruled out after initial workup consisting of chest radiography, electrocardiogram, and arterial blood gas”).  Definition of Bleeding: epidural hematoma, superficial hematoma, or persistent wound drainage. |

| **Study ID** | **Takahashi 2012** |
| --- | --- |
| **Design of the study** | Apparently retrospective cohort study |
| **Country** | Japan |
| **Period^1^** | 1990 – 1996 (group A)  2000 – 2011 (group B) |
| **Patients** | Inclusion criteria: Patients undergoing elective spine surgery  Exclusion criteria: Not reported |
| **Intervention/s** | Group A: No prophylaxis  Group B: Foot pump during surgery and for 24 or 48 h after surgery + elastic stockings for 1 week or until discharge. |
| **Outcome/s** | PE |
| **Follow up period** | Not reported |
| **Main results** | \|  \| Group A \| Group B \| \| --- \| --- \| --- \| \| Total patients included \| 541 \| 1434 \| \| Sex (cases, female) \| 205 \| 537 \| \| Age (years) \| 47,4 + 16,9 \| 57,0 + 17,5 \| \| Type of surgery   - Decompression - Fusion without instrumentation - Fusion with instrumentation \| 206  112  223 \| 920  52  462 \| \| DVT cases \| NR \| 0* \| \| PE cases \| 8 \| 3 \|   *We considered only symptomatic cases for purposes of meta-analysis in this case. In the case of symptomatic DVT, no direct data is provided, but it is stated that none of the 841 patients followed after surgery because of high D-dimer developed VTE. So, we assumed that none of this 841 patients developed DVT. |
| **Comments** | ***^1^For purposes of our systematic review, we only considered group B, as it was the only group receiving thromboprophylaxis***  Definition of DVT: Symptomatic DVT. No further description  Definition of PE: Symptomatic PE. No further description  This study also performed CT to a subgroup of group B (100 patients) to identify asymptomatic DVT and PE. In this subgroup, they identified 5 cases of asymptomatic DVT and 18 cases of asymptomatic PE (19 patients in total with DVT and/or PE). This data was not used for our meta-analysis |

| **Study ID** | **Tominaga 2015** |
| --- | --- |
| **Design of the study** | Retrospective cohort study |
| **Country** | Japan |
| **Period** | 2012 – 2013 |
| **Patients** | Inclusion criteria: Patients who had undergone spine surgery  Exclusion criteria: Patients having DVT preoperatively |
| **Intervention/s** | Mechanical methods, such as compression stockings and sequential pneumatic compression |
| **Outcome/s** | DVT, PE, Bleeding |
| **Follow up period** | 7 days |
| **Main results** | \| Total patients included \| 80 \| \| --- \| --- \| \| Sex (cases, female) \| 41 \| \| Age (years) \| 66,2 (22 – 85) \| \| Diagnosis   - Degenerative spondylolisthesis - Spondylolisthesis with lysis \|  \| \| DVT cases \| 20 \| \| PE cases \| 1 \| \| Bleeding cases \| 0 \| |
| **Comments** | Definition of DVT: According to ultrasonography (“All patients in this study had been screened by ultrasonography for DVTof the lower extremities 7 days after their surgery”)  Definition of PE: Computed tomography if symptoms were present  Definition of Bleeding: Epidural hematomas |

| **Study ID** | **Voth 1992** |
| --- | --- |
| **Design of the study** | Randomized controlled trial |
| **Country** | Germany |
| **Period** | Not reported |
| **Patients** | Inclusion criteria: Adults 40 years or older, undergoing surgical operation due to a prolapsed lumbar intervertebral disc.  Exclusion criteria: interfering concomitant medication, myocardial infarction or instable angina pectoris within the past six months, post-phlebitic leg syndrome, peripheral vascular disease, severe impairment of renal and/or hepatic function, severe hypertension,known bleeding abnormalities - such as haemophilia or thrombocytopenia -, symptoms of thyroid dysfunction and pregnancy. |
| **Intervention/s^1^** | Group A: Low molecular weight heparin (32 mg) + dihydroergotamine (0,5 mg) daily + placebo injection.  Group B: Unfractioned heparin (5000 U) + dihydroergotamine (0,5 mg) twice a day. |
| **Outcome/s** | DVT, PE, Bleeding |
| **Follow up period** | Mean duration of observation was 8,0 + 1,4 days for group A, and 7,6 + 1,6 days for group B |
| **Main results** | \|  \| Group A \| Group B \| \| --- \| --- \| --- \| \| Total patients included \| 87 \| 92 \| \| Sex (F:M) \| 49:38 \| 51:41 \| \| Age (years) \| 52,5 + 8,2 \| 52,8 + 9,1 \| \| Diagnosis \| Mentioned in inclusion criteria \| \| \| Surgical procedures (cases)   - Fenestration - Hemilaminectomy - Laminectomy - Sequestrotomy - Not mentioned \| 81  3  2  1  0 \| 85  4  1  0  2 \| \| DVT cases \| 4 \| 3 \| \| PE cases \| 0 \| 0 \| \| Bleeding cases \| 8 \| 7 \| |
| **Comments** | ***^1^ For purposes of this systematic review, data from both groups were considered as chemoprophylaxis.***  Definition of DVT: Compatible radiofibrinogen uptake-test  Definition of PE: Not clearly stated  Definition of Bleeding: Excessive intraoperative bleeding (surgeon’s subjective assessment), or wound hematoma during prophylaxis, or postoperative loss of blood in drains |

| **Study ID** | **Weber 2016** |
| --- | --- |
| **Design of the study** | Retrospective cohort study |
| **Country** | Australia |
| **Period** | 2008 – 2013 |
| **Patients** | Inclusion criteria: Patients who underwent elective posterior instrumented lumbar spinal fusion.  Exclusion criteria: |
| **Intervention/s** | Group A: Below the knee thromboembolic deterrent stockings and sequential calf-compression devices  Group B: Below the knee thromboembolic deterrent stockings and sequential calf-compression devices + low molecular weight heparin 4-6 hours postoperatively |
| **Outcome/s** | DVT, PE, Bleeding |
| **Follow up period** | Not clearly reported |
| **Main results** | \|  \| Group A \| Group B \| \| --- \| --- \| --- \| \| Total patients included \| 67 \| 40 \| \| Sex (% female) \| 58% \| \| \| Age (years) \| 58 + 12 (23 – 82) \| \| \| DVT cases \| 2 \| 0 \| \| PE cases \| 2 \| 0 \| \| Bleeding cases \| 0 \| 0 \| |
| **Comments** | Definition of DVT: Clinical and ultrasonography (“All patients were observed for clinical evidence of VTE and two-thirds were screened for DVT on day 4 or day 5 post-surgery with duplex ultrasound”)  Definition of PE: Clinical and imaging (“A computed tomography pulmonary angiogram was performed on all patients with clinical signs and/or symptoms suggestive of PE”)  Definition of Bleeding: Epidural hematoma |

| **Study ID** | **Wood 1997** |
| --- | --- |
| **Design of the study** | Randomized controlled trial |
| **Country** | USA |
| **Period** | 1994 – 1995 |
| **Patients** | Inclusion criteria: Adults patients undergoing major reconstructive spinal procedures, defined as those involving anterior or posterior (or both) thoracic, lumbar or thoracolumbar spine fusions or multilevel decompressions, or a combination of these.  Exclusion criteria: Patients undergoing cervical procedures, diskectomies, laminectomies, hardware removal, irrigation and debridements, posterior spine decompressions of only one level. Patients with a history of DVT or who preoperatively had such a medical risk for DVT as to require prophylaxis. Patients with a history of any of the following: pulmonary embolism, congestive heart failure, previous treatment with anticoagulants, or external conditions precluding the application of compression devices such as infection, neuropathy, or chronic venous stasis |
| **Intervention/s^1^** | Group A: Thigh-high compressions stockings (TED) + foot wraps  Group B: TED + sequential pneumatic compression wrap to the thigh |
| **Outcome/s** | DVT, PE, Bleeding |
| **Follow up period** | Clinical and ultrasonographic follow up until discharge, then 1 year of clinical follow up |
| **Main results** | \|  \| Group A \| Group B \| \| --- \| --- \| --- \| \| Total patients included \| 75 \| 59 \| \| Sex (% female) \| 48 \| 34 \| \| Age (years) \| 39,4 + 17,2 \| 39,6 + 18,5 \| \| Surgical procedures (cases, %)   - ALIF - ALIF/PSF - ASF - ASF/PSF - PSF - PSF/Decomp - PSF/PLIF/Decomp - Decomp - PSF/PLIF - SI Fusion - Other \| 1 (1,3%)  2 (2,6%)  9 (12%)  12 (16%)  28 (37,3%)  16 (21,3%)  1 (1,3%)  2 (2,6%)  0 (0%)  1 (1,3%)  3 (4%) \| 0 (0%)  0 (0%)  7 (11,9%)  16 (27,1%)  26 (44,1%)  7 (11,9%)  0 (0%)  2 (3,4%)  1 (1,7%)  0 (0%)  0 (0%) \| \| DVT cases \| 1 \| 0 \| \| PE cases \| 1 \| 0 \| \| Bleeding cases \| 0 \| 0 \|   ALIF: Anterior lumbar interbody fusion; PSF: Posterior spine fusion; PLIF: Posterior lumbar interbody fusion |
| **Comments** | ***^1^ For purposes of this systematic review, data from both groups were considered as mechanoprophylaxis***  Definition of DVT: According to Duplex ultrasonography (noncompressibility of the vein and vein distention, absent Doppler, lack of visible flow proximal to a noncompressible vein on more distal compression, or a visible clot within the vessel).  Definition of PE and Bleeding: Not clearly stated |

| **Study ID** | **Yamasaki 2017** |
| --- | --- |
| **Design of the study** | Retrospective cohort study |
| **Country** | Japan |
| **Period** | 2014 – 2016 |
| **Patients** | Inclusion criteria: Patients who underwent lumbar spine surgery.  Exclusion criteria: Not reported  Subjects were classified into four groups:   - Fracture group (F group) undergoing balloon kyphoplasty - Laminectomy group (La group) - Transforaminal lumbar interbody fusion (TLIF) group (T group) undergoing TLIF involving five or fewer segments - Long fusion group (Lo group) undergoing long spinal fusion involving six or more segments. |
| **Intervention/s** | Patients in whom DVT was found via preoperative lower limb ultrasonography received graduated compression stockings prior to surgery. Those without preoperative DVT received intermittent pneumatic compression during and after surgery.  Anticoagulant therapy was indicated for subjects with DVT:   - [1] who had unstable thrombosis advancing to the popliteal vein or - [2] who had unstable thrombosis involving multiple veins including the soleal veins, posterior tibial vein, and peroneal vein, excluding those with minimal thrombi only in a single vein.   Subjects categorized in Ref. [1] above began taking unfractionated heparin at one day postoperatively, and switched to a factor Xa inhibitor (edoxaban) at one week postoperatively. Subjects categorized in Ref. [2] above began taking a factor Xa inhibitor at one week postoperatively |
| **Outcome/s** | DVT |
| **Follow up period** | 7 days |
| **Main results** | \|  \| TOTAL \| F group \| La group \| T group \| Lo group \| \| --- \| --- \| --- \| --- \| --- \| --- \| \| Total patients included* \| 588 \| 101 \| 123 \| 230 \| 138 \| \| Sex (cases, female) \| 368 \| 74 \| 53 \| 117 \| 124 \| \| Age (years) \| Not reported \| 76,4 + 9,1 \| 64,6 + 17,7 \| 68,0 + 12,1 \| 71,7 + 6,8 \| \| Received anticoagulant therapy (chemical) \| 30 \| 0 \| 0 \| 8 \| 22 \| \| DVT cases   - Total - Preoperative - Postoperative \| 190  69  121 \| 37  27  10 \| 19  12  7 \| 59  14  45 \| 75  16  59 \|   *Data extracted from table 1. Sums are apparently incorrect in paper. |
| **Comments** | ***For purposes of meta-analysis, we considered this primary study as “mechanical prophylaxis”, because chemical thromboprophylaxis was initiated after the diagnosis of DVT. Also, because we aimed to analyze the incidence of thromboembolic events during or after surgery, patients with preoperative DVT were excluded from the analysis. F group was also excluded, as it does not meet our inclusion criteria. Therefore, we took in consideration for DVT incidence the total postoperative DVT cases of group La-T-Lo as numerator, and the total patients included in La-T-Lo groups as denominator.***  Definition of DVT: “Bilateral lower limb venous ultrasonography was performed in all subjects on the day before surgery, the day after surgery, and one week following surgery”. |

| **Study ID** | **Yang 2015 (A)** |
| --- | --- |
| **Design of the study** | Retrospective cohort study |
| **Country** | China |
| **Period** | 2013 – 2014 |
| **Patients** | Inclusion criteria: Patients who underwent spinal operations with complete medical records including patient number, sex, age, body weight, body height, regional distribution, admission date, hospital stay, occupation, lower extremity ultrasonography, DVT, spinal epidural hematoma, hypertension, diabetes, heart disease, surgical method, level and number of vertebrae fusion, duration of operation, blood loss, blood transfusion, length of incision, LMWH, prothrombin time activity (PTA), fibrinogen (FIB), thrombin time (TT), D-dimer, HDL (high density lipoprotein), LDL (low density lipoprotein), total cholesterol (TC), total bilirubin, direct bilirubin and indirect bilirubin (“However, some loss of the above records were accepted to include more patients “).  Exclusion criteria: Conservative treatment and percutaneous vertebroplasty (since the patients were discharged soon from hospital after percutaneous vertebroplasty), patients with pre-operative DVT, or patients who ever took anticoagulant such as warfarin, aspirin and clopidogrel during one week before hospital admission. |
| **Intervention/s** | Group A: Mechanical prophylaxis  Group B: Mechanical prophylaxis + Low molecular weight heparin (4100UI/day) from the 1st postoperative day to the 7th postoperative day. |
| **Outcome/s** | DVT, PE, Bleeding |
| **Follow up period** | Not reported |
| **Main results** | \|  \| Group A \| Group B \| \| --- \| --- \| --- \| \| Total patients included \| 86 \| 721 \| \| Sex (cases, female) \|  \|  \| \| Age (years, median (IQR)) \|  \|  \| \| DVT cases \| 7 \| 97 \| \| PE cases \| 0 \| 0 \| \| Bleeding cases \| 0 \| 2 \| |
| **Comments** | Definition of DVT: “Patients were examined by lower extremity ultrasonography pre- and postoperatively”  Definition of PE: Not reported  Definition of Bleeding: Spinal epidural hematoma |

| **Study ID** | **Yang 2015 (B)** |
| --- | --- |
| **Design of the study** | Retrospective cohort study |
| **Country** | China |
| **Period** | 2014 – 2015 |
| **Patients** | Inclusion criteria: Patients who underwent lumbar interbody fusion operations, with complete medical records including patient number, sex, age, body weight, body height, regional distribution, hospital stay, occupation, DVT, spinal epidural hematoma, hypertension, diabetes, heart disease, surgical method, level and number of vertebrae fusion, operation duration, blood loss, blood transfusion, incision length, prothrombin time activity (PTA), fibrinogen (FIB), thrombin time (TT), D-dimer, HDL (high-density lipoprotein), LDL (low-density lipoprotein), total cholesterol (TC), total bilirubin, direct bilirubin and indirect bilirubin, as well as VAS score in the first 3 postoperative days.  Exclusion criteria: Patients who did not undergo lumbar interbody fusion, had a prior DVT, or had been on anticoagulant therapy such as warfarin, aspirin, and clopidogrel prior to hospitalization. |
| **Intervention/s** | Prophylactic treatment with low-molecular-weight heparin at 4100 IU per day |
| **Outcome/s** | DVT, PE, Bleeding |
| **Follow up period** | Apparently 7 days |
| **Main results** | \| Total patients included \| 995 \| \| --- \| --- \| \| Sex (cases, female) \| 511 \| \| Age (years, median (IQR)) \| 50 (19) \| \| Levels of interbody fusion   - Single-level - Double-level - Three-level and above \| 731  218  46 \| \| DVT cases \| 223 \| \| PE cases \| 0 \| \| Bleeding cases \| 1 \| |
| **Comments** | Definition of DVT: “Lower extremity ultrasonography was routinely performed on all the patients on the 7th postoperative day”.  Definition of PE:Not reported  Definition of Bleeding: Spinal epidural hematoma |

| **Study ID** | **Yoshioka 2015** |
| --- | --- |
| **Design of the study** | Prospective cohort study |
| **Country** | Japan |
| **Period** | 2007 – 2012 |
| **Patients** | Inclusion criteria: patients undergoing degenerative spinal surgery  Exclusion criteria: Children younger than 18 years, patients treated with heparin for cerebral infarction or cardiac angina |
| **Intervention/s** | Compression stockings and intermittent pneumatic compression devices from the time of induction of general anesthesia to postoperative walking |
| **Outcome/s** | DVT, PE, Bleeding |
| **Follow up period** | 7 to 10 days |
| **Main results** | \|  \| Total \| Group 1 \| Group 2 \| Group 3 \| Group 4 \| Group 5 \| \| --- \| --- \| --- \| --- \| --- \| --- \| --- \| \| Total patients included \| 459 \| 108 \| 59 \| 93 \| 120 \| 79 \| \| Sex (cases, female) \| 198 \| 38 \| 26 \| 47 \| 43 \| 43 \| \| Age (years) \| 61,0 + 15,0 \| 62,0 + 12,4 \| 53,0 + 15,8 \| 61,0 + 16,1 \| 63,0 + 16,5 \| 64,0 + 13,8 \| \| Surgical approach   - Anterior - Posterior \| 16  567 \| 0  108 \| 16  43 \| 0  93 \| 0  120 \| 0  79 \| \| Surgical level   - Cervical - Thoracolumbar - Lumbar \| 167  93  199 \| 108  0  0 \| 59  0  0 \| 0  93  0 \| 0  0  120 \| 0  0  79 \| \| DVT cases \| 34 \| 3 \| 2 \| 8 \| 14 \| 7 \| \| PE cases \| 4 \| 0 \| 0 \| 2 \| 1 \| 0 \| \| Bleeding cases \| 0 \| 0 \| 0 \| 0 \| 0 \| 0 \|   Authors divided the population in five groups:   - Group 1: cervical degenerative disease treated with partial laminectomy or laminoplasty. - Group 2: cervical degenerative disease treated with instrumentation for spine fusion. - Group 3: thoracolumbar degenerative disease treated with instrumentation for spine fusion. - Group 4: lumbar spinal stenosis treated with posterior decompression. - Group 5: lumbar spondylolisthesis treated with 1-level posterior lumbar interbody fusion |
| **Comments** | Definition of DVT and PE: “All patients were examined by using a duplex ultrasonographical assessment of both lower extremities and with lung perfusion scintigraphy 7 to 10 days after surgery to ensure that asymptomatic thromboembolism was not missed. If PE was suspected or DVT was detected, the patient underwent multidetector computed tomographic (CT) venography. A definite diagnosis of DVT was made by using a duplex ultrasonographical assessment, and a definite diagnosis of PE was made by using multidetector CT venography”.  Definition of Bleeding: Postoperative hematoma  Of the cases of DVT reported, none was symptomatic. Of the cases of PE reported, 1 was symptomatic. |

| **Study ID** | **Zhi-jian 2011** |
| --- | --- |
| **Design of the study** | Prospective cohort study |
| **Country** | China |
| **Period** | 2004 – 2011 |
| **Patients** | Inclusion criteria: Patients undergoing posterior lumbar decompression surgery, classified as having high risk or the highest risk of VTE according to the 6th edition of ACCP Venous Thromboembolism Prevention Guideline and Levels of Thromboembolism Risk in Surgical Patients without Prophylaxis  Exclusion criteria:Haemorrhagic disease or other contraindications to Low Molecular Weight Heparin ( LMWH) |
| **Intervention/s** | Half dose of LMWH (2050 U) administered subcutaneously 6 hours after surgery, followed by a full dose (4100 U) once per day until discharge |
| **Outcome/s** | DVT, PE, Bleeding |
| **Follow up period** | Not clearly reported |
| **Main results** | \| Total patients included \| 78 \| \| --- \| --- \| \| Sex, female (% female) \| 49 (62,8%) \| \| Age (years) \| 62,4 + 10,6 \| \| Diagnosis (cases)   - Lumbar spondylolisthesis - Lumbar spinal stenosis - Lumbar disc herniation \| 10  55  13 \| \| Surgical procedure (cases)   - Lumbar decompression - Lumbar laminectomy and disectomy \| 65 (83,3%)  13 (16,7%) \| \| DVT cases \| 0 \| \| PE cases \| 0 \| \| Bleeding cases \| 0 \| |
| **Comments** | Definition of DVT and PE: According to Wells’ score  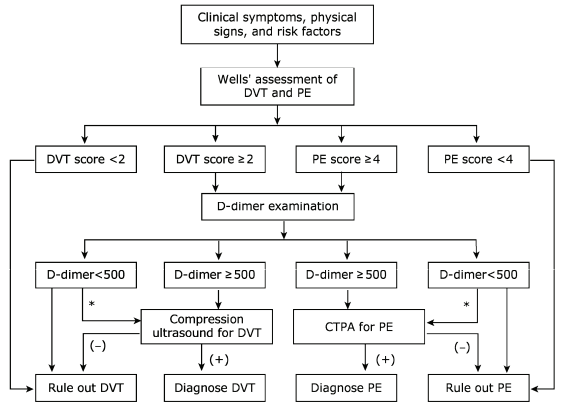  Definition of Bleeding: Acute bleeding fulfilling at least one of the following conditions: decrease in haemoglobin level by 20 g/L or more within 24 hours, transfusion requirement of two units or more of red blood cells, bleeding at a crucial site (including intracranial, spinal canal, intraocular, pericardial, retroperitoneal), bleeding at the surgical site requiring intervention, or intramuscular haemorrhage causing compartment syndrome. |

|  | **Chemoprophylaxis** | | | **Mechanical prophylaxis** | | | **Mixed thromboprophylaxis** | | |
| --- | --- | --- | --- | --- | --- | --- | --- | --- | --- |
| **Primary study** | **DVT** | **PE** | **Bleeding** | **DVT** | **PE** | **Bleeding** | **DVT** | **PE** | **Bleeding** |
| Akeda 2014 |  |  |  |  |  |  |  |  |  |
| Al-Dujaili 2012 |  |  |  |  |  |  |  |  |  |
| Cox 2014 |  |  |  |  |  |  |  |  |  |
| Dearborn 1999 |  |  |  |  |  |  |  |  |  |
| Du 2015 |  |  |  |  |  |  |  |  |  |
| Epstein 2005 |  |  |  |  |  |  |  |  |  |
| Epstein 2006 |  |  |  |  |  |  |  |  |  |
| Fawl 2017 |  |  |  |  |  |  |  |  |  |
| Ferree 1993 |  |  |  |  |  |  |  |  |  |
| Ferree 1994 |  |  |  |  |  |  |  |  |  |
| Gerlach 2003 |  |  |  |  |  |  |  |  |  |
| Gruber 1984 |  |  |  |  |  |  |  |  |  |
| Guo 2017 |  |  |  |  |  |  |  |  |  |
| Hamidi 2015 |  |  |  |  |  |  |  |  |  |
| Hohl 2015 |  |  |  |  |  |  |  |  |  |
| McLynn 2017 |  |  |  |  |  |  |  |  |  |
| Moayer 2016 |  |  |  |  |  |  |  |  |  |
| Nelson 1996 |  |  |  |  |  |  |  |  |  |
| Nicol 2009 |  |  |  |  |  |  |  |  |  |
| Pateder 2008 |  |  |  |  |  |  |  |  |  |
| Piasecki 2008 |  |  |  |  |  |  |  |  |  |
| Rokito 1996 |  |  |  |  |  |  |  |  |  |
| Schizas 2008 |  |  |  |  |  |  |  |  |  |
| Smith 1994 |  |  |  |  |  |  |  |  |  |
| Strom 2014 |  |  |  |  |  |  |  |  |  |
| Takahashi 2012 |  |  |  |  |  |  |  |  |  |
| Tominaga 2015 |  |  |  |  |  |  |  |  |  |
| Voth 1992 |  |  |  |  |  |  |  |  |  |
| Weber 2016 |  |  |  |  |  |  |  |  |  |
| Wood 1997 |  |  |  |  |  |  |  |  |  |
| Yamasaki 2017 |  |  |  |  |  |  |  |  |  |
| Yang 2015A |  |  |  |  |  |  |  |  |  |
| Yang 2015B |  |  |  |  |  |  |  |  |  |
| Yoshioka 2015 |  |  |  |  |  |  |  |  |  |
| Zhi-jian 2011 |  |  |  |  |  |  |  |  |  |
