# Supplementary material for: Thromboprophylaxis in elective spinal surgery: A protocol for systematic review
Source: Medicine (Baltimore). 2020 May 22;99(21):e20127. doi: 10.1097/MD.0000000000020127 (PMC7249943; doi:10.1097/MD.0000000000020127)
Supplement: Supplemental Digital Content [file medi-99-e20127-s004.docx]

**Appendix 3. Excludedstudies**

| **Study ID** | **Reason for exclusion** |
| --- | --- |
| **Aito 2002** | Different population |
| **Agnelli 1998** | Data not disaggregated |
| **Akhavan-Sigari 2014** | Different outcome |
| **Argwal 2009** | Different population |
| **Audibert 2005** | Other language |
| **Awad 2005** | Data not disaggregated |
| **Barnes 2004** | Different study design |
| **Becker 2005** | Other language |
| **Bono 2009** | Different study design |
| **Bryson 2012** | Different study design |
| **Burns 2005** | Different population |
| **Cabana 2000** | Other language |
| **Cain 1995** | Different intervention  Different study design |
| **Catre 1997** | Different study design |
| **Chen 2013** | Different population |
| **Cheng 2010** | Different study design |
| **Chiou-Tan 2003** | Different population |
| **Chitgopkar 2014** | Different study design |
| **Consortium for spinal cord medicine 1997** | Different population |
| **Christie 2011** | Different population |
| **Dazley 2012** | Different study design |
| **Deep 2001** | Different population |
| **Della Rocca 2013** | Different study design |
| **Desbordes 1993** | Other language |
| **Dhall 2013** | Different population |
| **Dickman 1992** | Different intervention |
| **Epstein 2015** | Different study design |
| **Epstein 2005** | Different study design |
| **Eskildsen 2015** | Different study design |
| **Eustratiades 1994** | Other language |
| **Fang 2011** | Different intervention |
| **Faciszewski 1995** | Different intervention |
| **Ferree 1993** | Different intervention |
| **Fineberg 2013** | Different intervention |
| **Flinn 1996** | Different population |
| **Frisbie 1981** | Different population |
| **Gephart 2012** | Different intervention |
| **Glotzbecker 2008** | Different study design |
| **Glotzbecker 2009** | Different study design |
| **Glotzbecker 2010** | Different study design |
| **Goldstein 2013** | Different intervention |
| **Gorman 2009** | Different population |
| **Goz 2014** | Different intervention |
| **Green 1982** | Different population |
| **Green 1988** | Different population |
| **Green 1990** | Different population |
| **Green 1991** | Different population |
| **Green 1992** | Different population |
| **Green 1994** | Different population |
| **Green 2003** | Different population |
| **Green 2003** | Different population |
| **Green 2005** | Different population |
| **Gunduz 1993** | Different population |
| **Guyatt 2012** | Different population |
| **Halim 2014** | Different population |
| **Harris 1996** | Different population |
| **Hebbeler 2004** | Different population |
| **Jacobs 2013** | Different population |
| **Jain 2014** | Different intervention |
| **Kannan 2002** | Different intervention |
| **Kao 2015** | Different intervention |
| **Kim 2011** | Different intervention |
| **Kim 2015** | Different population |
| **Koo 2014** | Data not disaggregated |
| **Kou 2002** | Different intervention |
| **Krasuski 1998** | Different population |
| **Kuhn 1991** | Other language |
| **Kulkarnis 1992** | Different population |
| **Kurtoglu 2004** | Different population |
| **Lawton 2005** | Different intervention |
| **Lee 2000** | Different intervention |
| **Leon 2005** | Different intervention |
| **Lohmann 2001** | Different population  Other language |
| **McClendon 2012** | Different intervention |
| **Marciniak 2012** | Different population |
| **Maxwell 2002** | Different population |
| **Merli 1993** | Different population |
| **Merli 1988** | Different population |
| **Miller 1983** | Different intervention |
| **Namboothiri 2012** | Different intervention |
| **Nicolaides 2006** | Different study design |
| **Nicolaides 2013** | Different study design |
| **Nillius 1980** | Different intervention |
| **North American Spine Society (NASS) 2009** | Different study design |
| **Oda 2000** | Different intervention |
| **Oglesby 2013** | Different intervention |
| **Oliveira 2014** | Different study design |
| **Otero-Fernández 2008** | Data not disaggregated |
| **Ozturk 2010** | Different intervention |
| **Paciaroni 2008** | Different population |
| **Papakostidis 2011** | Different population |
| **Platzer 2006** | Different population |
| **Ploumis 2009** | Different population |
| **Prestar 1992** | Other language |
| **Prothero 1994** | Different intervention |
| **Raj 2008** | Different study design |
| **Randelli 2013** | Different study design |
| **Rathore 2008** | Different population |
| **Rem 1989** | Other language |
| **Rojas-Tomba 2016** | Different intervention |
| **Rosner 2004** | Different study design |
| **Sansone 2010** | Different study design |
| **Scaduto 2003** | Different intervention |
| **Scavarda 1997** | Different intervention  Different study design  Other language |
| **Schoenfeld 2013** | Different intervention |
| **Schulte 2013** | Different intervention |
| **Schuste 2010** | Different study design |
| **Sebastian 2015** | Different intervention |
| **Sebastian 2016** | Different intervention |
| **Senders 2012** | Different intervention |
| **Silver 1971** | Different population |
| **Sing 2013** | Different study design |
| **Slavik 2007** | Different population |
| **Smith 2010** | Different intervention |
| **Smith 2004** | Different population |
| **Sobieraj 2012** | Different study design |
| **Sokolowski 2008** | Different intervention |
| **Spinal Cord Injury Thromboprophylaxis Investigators 2003** | Different population |
| **Spinal Cord Injury Thromboprophylaxis Investigators 2003** | Different population |
| **Steib 2011** | Different study design |
| **Stolke 1989** | Different intervention |
| **Struijk-Mulder  2010** | Different study design |
| **Teasell 2009** | Different population |
| **Tomaio 1998** | Different population |
| **Udén 1979** | Different intervention |
| **Uribe 2003** | Different intervention |
| **Vazquez 2013** | Different study design |
| **Wade 2000** | Different population |
| **Wade 2001** | Different population |
| **Wang 2015** | Different intervention |
| **Wang 2016** | Different outcome |
| **Weber 2014** | Different intervention |
| **Wen 1998** | Data not disaggregated |
| **West 1992** | Different intervention |
| **Worley 2008** | Different population |
| **Yang 2015** | Different intervention |
| **Yi 2006** | Different intervention |
| **Yonenobu 1991** | Different intervention |
| **Yoshioka 2013** | Same study as Yoshioka 2015 |
| **Yu 2011** | Other language |
