# Supplementary material for: Thromboprophylaxis in elective spinal surgery: A protocol for systematic review
Source: Medicine (Baltimore). 2020 May 22;99(21):e20127. doi: 10.1097/MD.0000000000020127 (PMC7249943; doi:10.1097/MD.0000000000020127)
Supplement: Supplemental Digital Content [file medi-99-e20127-s005.docx]

**Appendix 4. Risk of bias of included RCTs.**

| **Du 2015** | | |
| --- | --- | --- |
| Random sequence generation (selection bias) | Unclear risk | The specific method is not described |
| Allocation concealment (selection bias) | Low risk | The paper reports that the authors used ‘*sealed opaque envelopes’* |
| Blinding (performance bias and detection bias) | High risk | The study has an open design |
| Incomplete outcome data (attrition bias) | Low risk | There were no drop outs |
| Selective reporting (reporting bias) | Unclear risk | The study protocol is not available – apparently all results are reported |

| **Gruber 1984** | | |
| --- | --- | --- |
| Random sequence generation (selection bias) | Low risk | *‘A computer-produced randomization list was used.’* |
| Allocation concealment (selection bias) | Low risk | The paper reports that the authors used the *‘closed envelope technique’.* |
| Blinding (performance bias and detection bias) | Unclear risk | The study is reported as double-blind but no specific details are provided |
| Incomplete outcome data (attrition bias) | Unclear risk | In total 25 patients were randomized in each group; 5 patients in the placebo and 4 in the heparin-DHE group were admitted to the trial by error. Results are reported for the valid patients. There were no drop outs |
| Selective reporting (reporting bias) | Unclear risk | The study protocol is not available – apparently all results are reported |

| **Hamidi 2015** | | |
| --- | --- | --- |
| Random sequence generation (selection bias) | Unclear risk | The specific method is not described |
| Allocation concealment (selection bias) | Unclear risk | It is not reported how the sequence was concealed, if so |
| Blinding (performance bias and detection bias) | High risk | The study has an open design |
| Incomplete outcome data (attrition bias) | Low risk | There were no drop outs |
| Selective reporting (reporting bias) | Unclear risk | The study protocol is not available – apparently all results are reported |

| [**Nelson 1996**](https://www.ncbi.nlm.nih.gov/pubmed/?term=Nelson%20LD%20Jr%5BAuthor%5D&cauthor=true&cauthor_uid=8884705) | | |
| --- | --- | --- |
| Random sequence generation (selection bias) | Unclear risk | The specific method is not described |
| Allocation concealment (selection bias) | Unclear risk | It is not reported how the sequence was concealed, if so |
| Blinding  (performance bias and detection bias) | High risk | The study has an open design |
| Incomplete outcome data (attrition bias) | Unclear risk | The authors did not performed an ITT analysis |
| Selective reporting (reporting bias) | Unclear risk | The study protocol is not available – apparently all results are reported |

| [**Rokito**](https://www.ncbi.nlm.nih.gov/pubmed/?term=Rokito%20SE%5BAuthor%5D&cauthor=true&cauthor_uid=8779018) **1996** | | |
| --- | --- | --- |
| Random sequence generation (selection bias) | Unclear risk | The specific method is not described |
| Allocation concealment (selection bias) | Unclear risk | It is not reported how the sequence was concealed, if so |
| Blinding (performance bias and detection bias) | High risk | The study has an open design |
| Incomplete outcome data (attrition bias) | Low risk | There were no drop outs |
| Selective reporting (reporting bias) | Unclear risk | The study protocol is not available – apparently all results are reported |

| **Voth 1992** | | |
| --- | --- | --- |
| Random sequence generation (selection bias) | Unclear risk | The specific method is not described |
| Allocation concealment (selection bias) | Unclear risk | It is not reported how the sequence was concealed, if so |
| Blinding  (performance bias and detection bias) | Unclear risk | The study is reported as double-blind but no specific details are provided |
| Incomplete outcome data (attrition bias) | Unclear risk | The authors did not performed an ITT analysis |
| Selective reporting (reporting bias) | Unclear risk | The study protocol is not available – apparently all results are reported |

| **Wood 1997** | | |
| --- | --- | --- |
| Random sequence generation (selection bias) | Unclear risk | The specific method is not described |
| Allocation concealment (selection bias) | Unclear risk | It is not reported how the sequence was concealed, if so |
| Blinding  (performance bias and detection bias) | High risk | The study has an open design |
| Incomplete outcome data (attrition bias) | Unclear risk | The authors did not performed an ITT analysis |
| Selective reporting (reporting bias) | Unclear risk | The study protocol is not available – apparently all results are reported |
